# Memory Elicited by Courtship Conditioning Requires Mushroom Body Neuronal Subsets Similar to Those Utilized in Appetitive Memory

**DOI:** 10.1371/journal.pone.0164516

**Published:** 2016-10-20

**Authors:** Shelby A. Montague, Bruce S. Baker

**Affiliations:** Howard Hughes Medical Institute Janelia Research Campus, Ashburn, Virginia, United States of America; Lancaster University, UNITED KINGDOM

## Abstract

An animal’s ability to learn and to form memories is essential for its survival. The fruit fly has proven to be a valuable model system for studies of learning and memory. One learned behavior in fruit flies is courtship conditioning. In *Drosophila* courtship conditioning, male flies learn not to court females during training with an unreceptive female. He retains a memory of this training and for several hours decreases courtship when subsequently paired with any female. Courtship conditioning is a unique learning paradigm; it uses a positive-valence stimulus, a female fly, to teach a male to decrease an innate behavior, courtship of the female. As such, courtship conditioning is not clearly categorized as either appetitive or aversive conditioning. The mushroom body (MB) region in the fruit fly brain is important for several types of memory; however, the precise subsets of intrinsic and extrinsic MB neurons necessary for courtship conditioning are unknown. Here, we disrupted synaptic signaling by driving a shibire^ts^ effector in precise subsets of MB neurons, defined by a collection of split-GAL4 drivers. Out of 75 lines tested, 32 showed defects in courtship conditioning memory. Surprisingly, we did not have any hits in the γ lobe Kenyon cells, a region previously implicated in courtship conditioning memory. We did find that several γ lobe extrinsic neurons were necessary for courtship conditioning memory. Overall, our memory hits in the dopaminergic neurons (DANs) and the mushroom body output neurons were more consistent with results from appetitive memory assays than aversive memory assays. For example, protocerebral anterior medial DANs were necessary for courtship memory, similar to appetitive memory, while protocerebral posterior lateral 1 (PPL1) DANs, important for aversive memory, were not needed. Overall, our results indicate that the MB circuits necessary for courtship conditioning memory coincide with circuits necessary for appetitive memory.

## Introduction

In order to adapt to a changing environment, an animal must be able to respond to sensory cues. In general, such cues will be inherently attractive, repellant, or neutral to the animal. Being able to remember a cue relative to its context is potentially advantageous for an animal, as the organism can then respond to a repeat presentation of the cue in an appropriate manner.

Several models exist for the study of learning and memory. One powerful model system is provided by the fruit fly *Drosophila melanogaster*. Fruit flies display a variety of behaviors, and they can be manipulated using many different genetic tools. For example, by using these genetic tools to manipulate the functions of identical subsets of neurons across many individuals and then observing changes in behavior, the roles of particular neurons in learning and memory can be elucidated.

In the fruit fly, one major memory center in the brain is a region called the mushroom bodies (MBs) [1–4]. Both appetitive memory, the association between a cue and a reward, and aversive memory, the association between a cue and a punishment, require MB activity [4]. However, researchers have found that, while similar sets of mushroom body neurons are important for both appetitive and aversive memory, different extrinsic neurons are important in each memory type [5–16]. For example, protocerebral posterior lateral 1 (PPL1) dopaminergic neurons (DANs) are important for aversive conditioning [7, 8, 14–16], whereas protocerebral anterior medial (PAM) DANs are important for appetitive memory [5, 13, 15, 16, 17].

Courtship conditioning is one learning and memory task in fruit flies. While most learning and memory tasks teach approach or avoidance by pairing two individual, simple stimuli, such as an odor paired with an electric shock, courtship conditioning is unique because it teaches a complex form of learning, reduction of courtship behavior, using a natural stimulus, another fruit fly [18–20]. Briefly, when a naïve male fly is paired with an unreceptive female fly, he fails to copulate and learns to stop courting her. The male retains the memory of failed copulation [21, 22], and, after a one-hour training period, he displays decreased courtship towards a new female for at least two hours [19]. Courtship conditioning is not explicitly appetitive memory; the male is not forming an association between a stimulus and a reward. However, it also is not explicitly aversive memory. The male does form a negative association between a female fly and a lack of copulation, but the stimulus involved, specifically the female fly and her associated sensory cues, is innately attractive to a naïve male.

The mushroom bodies are required for memory of courtship conditioning, hereafter called courtship memory. Male fruit flies with reduced or completely ablated MBs learn to stop courting unreceptive females, but they lose the memory within thirty minutes [23, 24]. Courtship memory also requires certain proteins and genes to be functioning in the MBs, including fru^M^, CAMKII, and *Orb2* [21, 25–29]. While several studies have examined changes in memory caused by disrupting these or other genes, few studies have examined changes in memory caused by disrupting synaptic signaling. Those studies have targeted either the MBs as a whole or particular subsets of neurons without screening across the entire MBs and their extrinsic neurons [21, 30].

In the fruit fly, the bipartite GAL4-UAS system can be used to target the expression of a transgene in specific sets of cells [31]. However, most GAL4 lines have broad expression patterns [32, 33]. To improve the specificity of this system, the derivative split-GAL4 system was developed [34]. The split-GAL4 system breaks the GAL4 sequence into two pieces each driven by a unique enhancer, both of which must be expressed in a cell to reconstitute a functional GAL4 protein. This system was used to create a comprehensive set of mushroom body split-GAL4 lines [35]. These lines include those expressed in subsets of neurons both intrinsic to the MBs, the Kenyon cells (KCs), and extrinsic to the MBs, including output neurons and modulatory DANs [35]. Using these lines, several recent studies have examined the roles of subsets of MB neurons in aversive and appetitive learning and memory [11, 14–17], but none have studied courtship conditioning. The introduction of the split-GAL4 system allows, for the first time, a comprehensive and systematic examination of the roles of small subsets of MB neurons in courtship conditioning.

To examine the roles of subsets of MB neurons in learning and memory during courtship conditioning, we screened 75 mushroom body split-GAL4 lines in combination with a shibire^ts^ effector to disrupt synaptic signaling. Shibire^ts^ is a dynamin transgene that disrupts the recycling of synaptic vesicles when placed at the restrictive temperature (>29°C), allowing a short-term block of synaptic signaling [36]. Changes in courtship behavior were measured to test for changes in learning and memory. While the majority of lines did learn to stop courting, roughly half of all lines lacked courtship memory. Lines lacking memory are expressed in several neuron types, including KCs, mushroom body output neurons (MBONs), and PAM DANs. To verify the roles of particular subsets in courtship memory, we repeated experiments for lines of interest. Only one line expressing in the γ KCs was needed for memory in our screen, a finding that surprised us since this region has a reported role in courtship memory [21, 27, 28, 30, 37]. Even though activity of γ KCs was not needed for courtship conditioning, several lines extrinsic to the γ lobes, including MBONs and PAM DANs, were needed for courtship memory. Several lines expressing in or innervating the α/β and α’/β’ lobes also were needed for courtship memory. To determine whether neuronal subsets needed for courtship memory were more similar to subsets needed for appetitive memory or for aversive memory at the neuronal level, we then compared the neuron types identified in our study to neuron types required for each type of memory as reported by previous studies. We were surprised to find that most of the MB neurons required for courtship memory overlapped with neurons required for appetitive memory. Taken together, our experiments suggest that many MB neuron subsets are important in the formation of courtship memory and that courtship memory and appetitive memory share neuronal circuits.

## Results

### Screening Mushroom Body Neuronal Subsets in a Courtship Conditioning Assay

**Fig 1:**
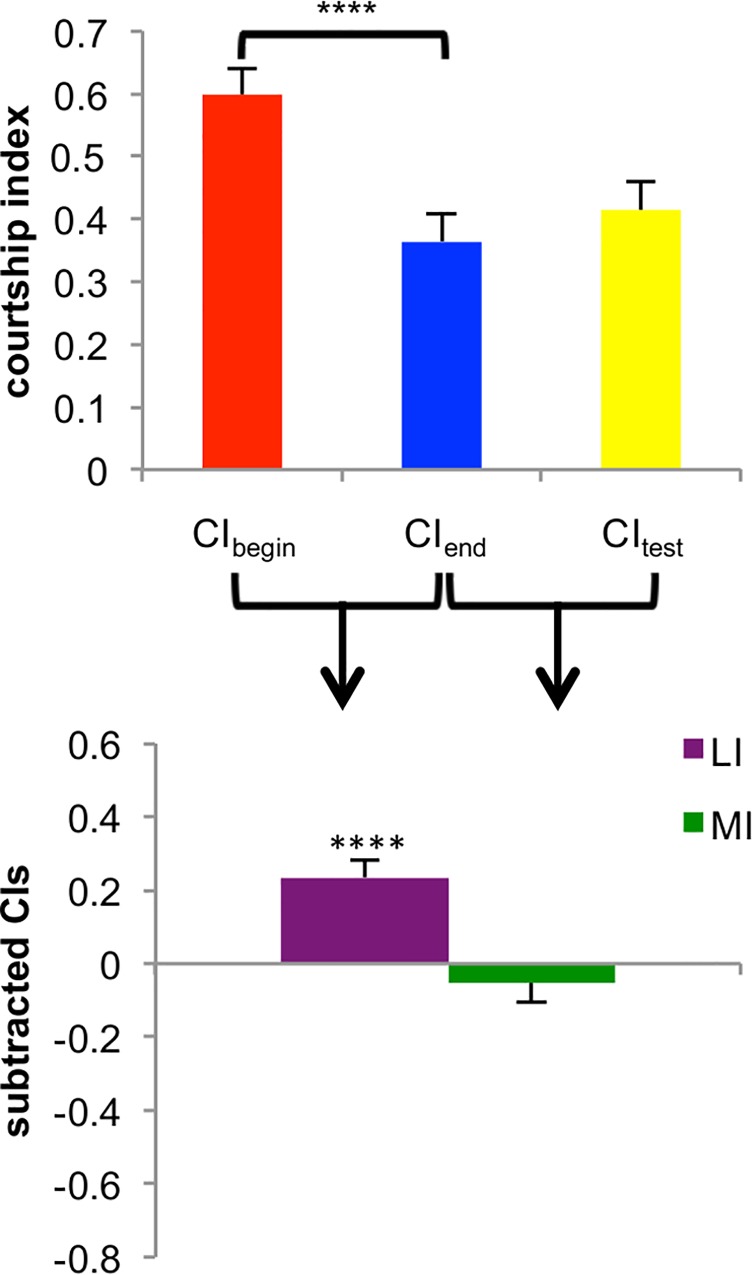
Calculation of Learning and Memory Indices. For all experiments, a courtship index (CI) was calculated as the percent of time spent performing courtship behaviors in three different time periods: the ten minutes at the beginning of training (CI_begin_), the ten minutes at the end of training (CI_end_), and the ten minute test period (CI_test_). In this example, the CIs for each of these three periods are plotted for wild-type DL flies tested at 25C. To generate the learning index (LI), we subtract CI_begin_−CI_end_. A significantly positive LI indicates that learning occurred. To generate the memory index (MI), we subtract CI_end_−CI_test_. An MI close to zero indicates that memory occurred, and a significantly negative MI indicates a lack of memory. In these flies, significant learning occurred, but there was not a significant change between the CI_end_ and the CI_test_, indicating memory also occurred. Significance is determined using a one-sided Wilcoxon signed rank test; learning significance is a comparison between CI_begin_ and CI_end_, and memory significance is a comparison between CI_end_ and CI_test_. ****, p < .0001. Error bars are SEM, n = 48.

To test the role of the mushroom bodies in learning and memory during courtship conditioning, we inactivated subsets of intrinsic and extrinsic MB neurons. To do this, we blocked synaptic transmission by crossing a shibire^ts^ effector with each of 75 mushroom body (MB) split-GAL4 driver lines. These split-GAL4; UAS-shi^ts^ males were screened for changes in courtship behavior during courtship conditioning assays performed similarly to previous experiments [19, 28, 29, 38]. The male was paired with a mated female (the trainer) for one hour to teach him to reduce courtship. We kept track of individual males, moving each to a new courtship chamber for a thirty minute wait following the training period. At the end of this wait, during which memory can decay, the barrier separating the male and a decapitated virgin female (the tester) was removed, and courtship was recorded for ten minutes. For each fly, the courtship index (CI) at the end of training was subtracted from the CI at the beginning of training to calculate a learning index (LI = CI_begin_−CI_end_; a positive LI means the male courted less at the end of the training period and thus he learned). The CI during the test period was subtracted from the CI at the end of training to calculate a memory index (MI = CI_end_−CI_test_; a value close to zero means the fly reduced courtship towards the tester and thus he remembered the training) (Fig 1, S1 Table).

Many of the 75 split-GAL4 lines tested express in overlapping sets of neurons, and, in some cases, not all lines expressing in a particular neuron type were consistently hits or not hits. There are several reasons why these differences may occur. First, while many lines express in overlapping neuron types, most of these lines are not expressed in identical sets of neurons [35]. It is possible that there are different requirements for the different neuron populations. Second, even for lines that express in identical populations, the split-GAL4’s often have different expression levels in those neurons [11], possibly causing differences in the level of expression of the shibire^ts^ effector. Third, each line has a slightly different genetic background. Different enhancer elements were combined to create each split-GAL4, so even two lines expressing in overlapping neuron populations may have differences in the expression of their enhancers during development or in the adult fly. Fourth, all of the MB split-GAL4 lines were imaged in adult female flies [35]. Because we are testing courtship conditioning learning and memory, we are using male flies. It is possible that some lines have sexually dimorphic expression; the male flies may have different expression patterns than those reported, and blocking activity in the sexually dimorphic neurons could be causing changes in behavior. Despite these caveats, we identified many neurons as potential candidates for having a role in courtship memory, and we verified the roles of some of these lines in re-tests.

### Intrinsic and Extrinsic Mushroom Body Neurons are not Needed to Learn Courtship Conditioning

**Fig 2:**
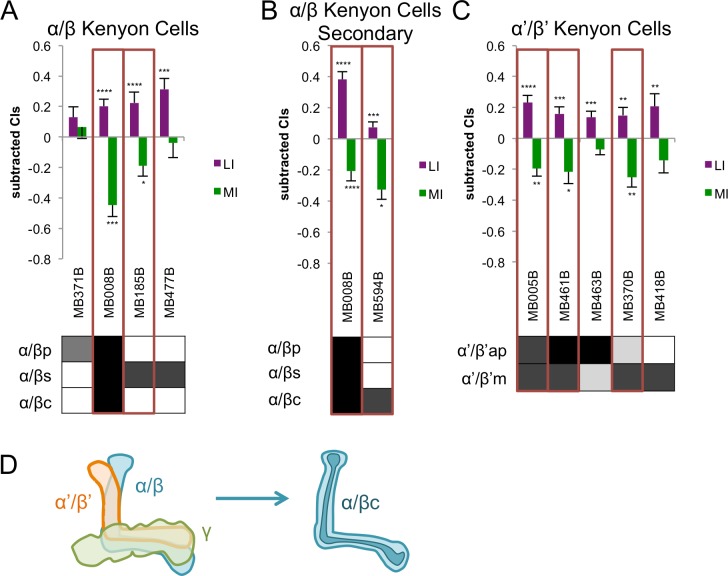
Courtship Conditioning Learning and Memory in Kenyon Cell Lines. Learning index (LI) and memory index (MI) for A. α/β Kenyon cell lines, B. secondary α/β Kenyon cell lines, and C. α’/β’ Kenyon cell lines. Lines identified as courtship memory hits are boxed in red. Expression patterns are directly below the LI and MI for each line. Shading indicates relative levels of expression in each neuron type as reported in [35]. Significance is determined using one-sided Wilcoxon signed ranks tests with Benjamini-Hochberg corrections on primary screen data. *, p < .05; **, p < .01; ***, p < .001; ****, p < .0001. Error bars are SEM, n = 19–46. D. Diagrams of the mushroom bodies and the general area of expression of the α/βc neurons [35].

**Fig 3:**
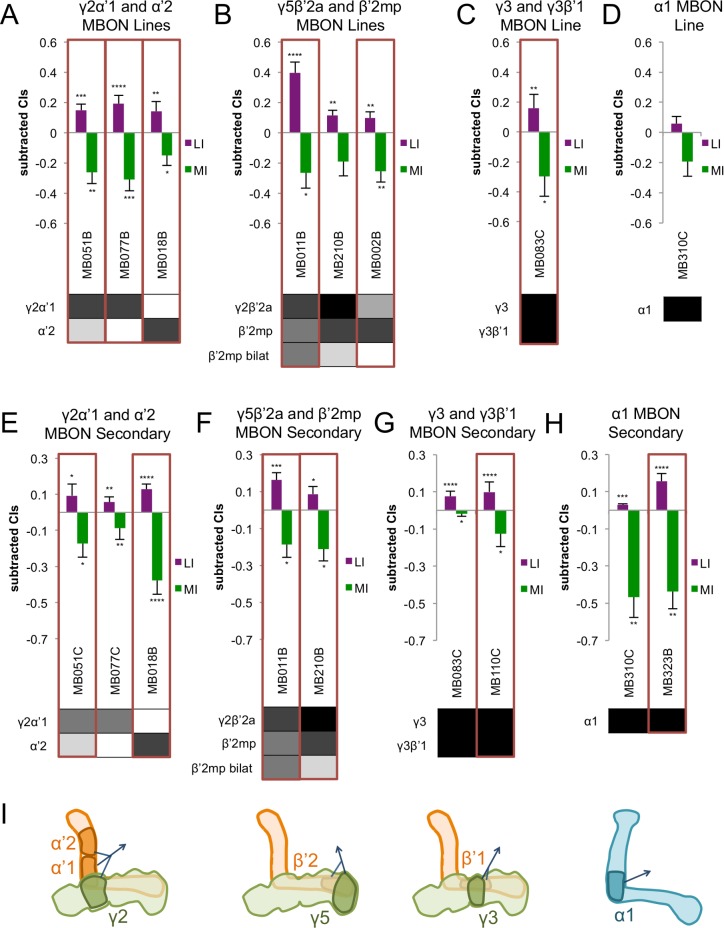
Courtship Conditioning Learning and Memory in MB Output Neurons. Learning index (LI) and memory index (MI) for A. xγ2α’1 and α’2 MBON lines, B. γ2β’2a and β’2mp MBON lines, C. γ3 and γ3β’1 MBON lines, and D. α1 MBON line. Lines identified as courtship memory hits are boxed in red. Expression patterns are directly below the LI and MI for each line. Shading indicates relative levels of expression as reported in [35]. Significance is determined using one-sided Wilcoxon signed rank tests with Benjamini-Hochberg post-hoc corrections. *, p < .05; **, p < .01; ***, p < .001; ****, p < .0001. Error bars are SEM, n = 9–24. Secondary screening for E. γ2α‘1 and α‘2 MBON lines, F.γ2β’2a and β’2mp MBON lines, G. γ3 and γ3β’1 MBON lines, and H. α1 MBON lines. Lines identified as courtship memory hits are boxed in red. Expression patterns are directly below the LI and MI for each line. Shading indicates relative levels of expression as reported in [35]. Significance is determined using one-sided Wilcoxon signed rank tests. *, p < .05; **, p < .01; ***, p < .001; ****, p < .0001. Error bars are SEM, n = 8–46. I. Diagram of the mushroom bodies and the sites of innervation of the γ2α‘1 and α‘2 MBONs, γ3 and γ3β’1 MBONs, γ5β’2a and β’2mp MBONs, and α1 MBONs [35].

Out of the 75 lines tested, only 8 did not have significant learning (Kenyon cells (KCs): MB371B (Fig 2) and MB419B (S2 Fig); protocerebral anterior medial (PAM) dopaminergic neurons (DANs): MB047B (S6 Fig); protocerebral posterior lateral 1 (PPL1) DANs: MB438B and MB439B (S6 Fig); mushroom body output neurons (MBONs): MB310C (Fig 3), MB298B, and MB434B (S4 Fig, p>0.05). Five of these eight lines were just slightly above the threshold for significance (MB419B, MB371B, MB298B, MB310C, and MB047B; p≤0.10). Two of the remaining three lines (MB438B and MB439B) both express in PPL1 neurons, but other lines expressing in these neurons have significant learning when used to drive expression of *shibire*^*ts*^ (i.e. MB065B and MB502B, S6 Fig). This discrepancy in learning indicates that the lack of learning likely is not because of disrupting synaptic signaling in these neurons. These results indicating that the MBs are not needed for learning in courtship conditioning are consistent with previous studies [23, 24]. We focus the rest of our analysis and conclusions on the roles of MB neuronal subsets in the memory of courtship conditioning.

### Intrinsic and Extrinsic Neurons are Needed to Remember Courtship Conditioning

To determine which neuronal subsets are needed to remember courtship conditioning, we set three criteria to call a line a hit: 1. The line must have significant learning, 2. The line must have a significant lack of memory, and 3. The average CI_begin_ must be greater than 0.1. We included the third criterion because, when the initial CI is low, it is difficult to use reductions compared to this CI to conclude that flies do not learn or remember. This criterion excluded only one line as a hit in our primary screen, MB080C, expressing in α2sc MBONs (S4 Fig).

Using these criteria, 32 out of 75 lines lacked courtship memory, a hit rate of almost 50%. Such a high hit rate is likely due to our screen being biased; we are selectively testing neurons in a brain region necessary for learning and memory in general and for courtship conditioning in particular. We could have false positives, either because we are affecting some other aspect of courtship or because we are affecting the neurons in a way not directly related to courtship memory. We do not believe we are affecting courtship behavior in general because we observed each fly directly to calculate CIs, and we did not note any obvious changes in behavior. Non-specific effects on neurons are not likely because of the specificity of the split-GAL4 lines, but this is still a possibility. To help test this possibility, we performed control tests for some lines by testing the same genotype, the split-GAL4 crossed with the shibire^ts^ effector, at a permissive temperature. For our analysis, we grouped the lines based on their published expression patterns [35].

### Subsets of Kenyon Cells are Needed for Courtship Memory

Of the 16 Kenyon cell (KC) lines tested for courtship memory, 7 were hits in the primary screen. These hits included two lines expressing in α/β neurons, three lines expressing in α’/β’ neurons, and two broadly expressing lines.

The γ lobes of the MBs contain the most fruitless^M^ (fru^M^) -positive KCs [37, 39, 40]. fru^M^ is a male-specific transcription factor important for courtship behavior [37, 39]. The γ lobes are also important for the formation of both appetitive and aversive memories [7, 9, 10, 41–43]. Of the three lines we tested with specific expression in all or part of the γ lobe (MB419B, MB009B, MB131B), none of them were hits in the primary screen (S2 and S3 Figs). Because of the γ lobes’ reported role in courtship memory [21, 27, 28, 30, 37], we performed secondary experiments using 3 lines specifically expressed in the γ lobes, including one line not tested in the primary screen (MB607B). These secondary experiments were performed identically to the primary screen experiments; they constitute genetic replicas, with flies collected from a new cross between the split-GAL4 of interest and the same shibire^ts^ effector. One of these lines, expressed in γ dorsal lobes (MB419B), was a hit (S2 and S3 Figs). The new line tested, MB607B, was not a hit because it did not learn, and MB131B was not a hit because of low CI_begin_ (S2 Fig). Two of these lines, MB419B and MB607B, were also tested at permissive temperature. MB419B showed significant learning and did not show a lack of memory, as expected for a control line (LI: p<0.05; MI: p>>0.05; S1 Table), but MB 607B did not have significant learning at the permissive temperature (LI: p>0.05; S1 Table). The differences in hits could be because the lines are expressed in different neuronal subsets, because they have different levels of expression in the same neurons, or because of their different genetic backgrounds, as mentioned above. Because MB607B also did not show significant learning at the permissive temperature, it is likely that the last possibility, differences in genetic background, explain the difference in hits. These results suggest that synaptic signaling in the γ lobes may not be necessary for courtship memory.

The α/β lobes of the MBs may be needed for courtship memory. The α/β core (α/βc) contains fru^M^-positive KCs [37, 40] and all or part of the α/β lobes has a role in courtship memory [25, 29, 30]. Additionally, α/β KCs have been implicated more broadly in multiple forms of aversive olfactory memory and are thought to be important for memory retrieval [7, 9, 41, 43, 44]. In particular, the α/βc neurons are important for appetitive olfactory memory whereas the α/β surface (α/βs) neurons are important for aversive olfactory memory [45]. Of four lines we tested with specific expression in these lobes, two lines lacked memory (MB008B and MB185B, Fig 2 and S3 Fig). Lines expressing in α/βs neurons had conflicting results (MB185B was a hit, MB477B was not, Fig 2); these lines may not be expressed in identical subsets of neurons, as mentioned above. The other hit, MB008B, is expressed throughout the α/β lobes, and it was verified as a memory hit in secondary screening (Fig 2 and S3 Fig). We also tested a line expressing only in α/βc neurons, MB594B, in secondary screening, and this line was a hit (Fig 2 and S3 Fig). When tested at permissive temperature, the MB594B line showed significant learning and a significant loss of memory (LI: p<0.01; MI: p<0.05; S1 Table). These results show that the α/βc neurons are needed to remember courtship conditioning, consistent with possible overlap between courtship memory and appetitive memory neuronal circuits.

The third set of MB lobes, the α’/β’ lobes, were not thought to be involved in courtship memory based on studies using GAL4 lines to restrict activity of Orb2, an RNA binding protein, or fru^M^ in subsets of mushroom body neurons [21, 37]. However, activity of α’/β’ neurons is important for both appetitive and aversive memory immediately after training and is believed to be important for memory consolidation [44, 46]. Surprisingly, lines expressing in these lobes were the greatest proportion of hits in our primary screen. Three of five lines specifically expressed in the α’/β’ lobes were hits (MB005B, MB461B, and MB370B, Fig 2 and S3 Fig). The memory index for one of the two lines that were not hits, MB418B, was just above the threshold for significance (p = .06). The difference between lines that are hits and those that are not could be a result of expression in different subsets of neurons, different levels of expression in the same neurons, or different genetic backgrounds. To verify the role of α’/β’ KCs, we tested MB005B and MB461B in secondary assays. While both lines had significant learning and a significant lack of memory, neither were hits because of low levels of courtship at the beginning of training (S2 and S3 Figs). Both of these lines were also tested at permissive temperature; both lines had much high levels of initial courtship (CI_begin_ = 0.71 and 0.44, respectively; S1 Table), both showed significant learning (LI: p<0.05 for both; S1 Table), and neither line showed a lack of memory (MI: p>0.05 for both; S1 Table). These control experiments indicate that these two lines may not perform courtship well at the restrictive temperature used in this screen. The α’/β’ lobes may play a role in courtship memory similar to their role in other types of memory, but additional experiments are necessary to verify that our hits are a result of disrupting synaptic activity in these neurons.

Since multiple subsets of KCs were courtship memory hits, we concluded that disrupting synaptic signaling throughout the MBs might also inhibit memory. However, the two most broadly-expressing lines, MB010B and MB152B, were not hits (S2 and S3 Figs). By contrast, two other lines expressing in all but one region of the MBs, MB364B, which does not express in the α’/β’ lobes, and MB417B, which does not express in the γ lobe, were memory hits (S2 and S3 Figs). It is possible that these two lines were hits because they have stronger effects on the α/β lobes, the region where they both express, or because decreasing activity in subsets of the MBs has a stronger overall effect on courtship memory than decreasing activity in the entire MBs.

### Several Output Neurons are Needed for Courtship Memory

Of 25 output neuron (ON) lines tested, 11 were courtship memory hits. These hits covered 11 different neuron types, including MBONs projecting from regions throughout the MB lobes.

Two different lines expressing in γ2α’1 MBONs, MB051B and MB077B, were required for memory (Fig 3 and S5 Fig). These same split-GAL4 lines crossed with a similar shibire^ts^ effector are important in appetitive odor memory and appetitive ethanol memory [11]. One of these lines, MB051B, also expresses in α’2 MBONs, and another line expressing in α’2 MBONs, MB018B, was also a hit (Fig 3). Two additional α’2 MBON lines, MB093C and MB082C, were not hits; however, these lines have stronger relative expression levels in the α3 MBONs (S4 Fig). As mentioned above, expression in different neuronal subsets could be one reason why some lines are hits while others are not, and the expression in the α3 MBONs may be the reason these lines are not hits. In secondary assays, we tested three lines expressing in γ2α’1 and α’2 MBONs: two not tested in primary screening (MB051C and MB077C) and one previously tested (MB018B). MB077C was not a hit because of a low level of courtship at the start of training; the other lines, MB051C, which expresses in both γ2α’1 and α’2 ONs, and MB018B, which expresses in α’2 MBONs, were hits (Fig 3 and S5 Fig). These three lines were also tested at permissive temperature. Unfortunately, none of these controls matched the expected behavior of controls. MB077C had a much higher initial level of courtship (CI_begin_ = 0.43, S1 Table) but also had a significant lack of memory (LI: p<0.001; MI: p<0.05; S1 Table). MB018B also showed a significant lack of memory (LI: p<0.001; MI: p<0.001; S1 Table), and MB051C did not show significant learning (LI: p>0.05; S1 Table). Taken together, we conclude that, while there is evidence to support a role of the γ2α’1 and α’2 MBONs in remembering courtship conditioning, our control data indicate that genetic background may cause some of the problems with memory. Additional experiments would be required to confirm a role of the γ2α’1 and α’2 MBONs.

The γ5β’2a and β’2mp output neurons (also called the M4/M6 clusters) may be involved in courtship memory because these neurons are important for both appetitive and aversive olfactory memory [47]. These neurons are also needed for appetitive ethanol memory and appetitive visual memory [11]. Two lines expressing in these neurons, MB011B and MB002B, were courtship memory hits (Fig 3 and S5 Fig), but two other lines, MB210B (Fig 3) and MB074C (S4 Fig), were not hits. MB210B had a memory index close to significance (p = 0.07). MB074C is also expressed in the β2β’2a output neurons, and another line expressed specifically in these neurons, MB399B, also was not a hit (S4 Fig). These differences in hits could be due to different levels of expression in the same neurons or due to expression in different neuronal subsets, respectively. We tested two of these lines, MB011B and MB210B, in secondary assays, and both were hits, confirming a role for these neurons in courtship memory (Fig 3 and S5 Fig). When tested at permissive temperature, both of these lines showed significant learning and no significant lack of memory (LI: p<0.05; MI: p>0.10; S1 Table). Taken together, the requirement of the γ5β’2a and β’2mp output neurons provides further evidence for the overlap between courtship memory and appetitive memory circuits.

Two different MBON neuron types project into another region of the γ lobe, γ3 and γ3β’1 MBONs. In our primary screen, we only had one line that expressed in these neurons, and this line was a memory hit (MB083C, Fig 3 and S5 Fig). We repeated assays with this line and tested an additional line, MB110C, in secondary screening. MB083C was not a hit in this secondary retest because of low levels of courtship, but MB110C was needed for courtship memory (Fig 3). When tested at permissive temperature, MB083C did not show significant learning (LI: p = 0.5; S1 Table), but MB110C showed significant learning and no significant lack of memory (LI: p<0.01; MI: p>0.3; S1 Table). These results indicate that the γ3 and/or γ3β’1 MBONs are required for courtship memory, but additional tests are needed to confirm this requirement.

We identified lines lacking courtship memory that express in the α/β KCs and in the PAM α1 neurons (see below). Since these neurons could form a circuit with α1 MBONs, we performed secondary assays with α1 MBON lines even though MB310C, the only line expressed in these neurons, was not a hit in the primary screen (Fig 3 and S5 Fig). We tested MB310C and an additional line, MB323B. MB310C again was not a memory hit, because of low levels of initial courtship, but MB323B was needed for memory (Fig 3 and S5 Fig). When tested at permissive temperature, both of these lines showed significant learning (LI: p<0.01 for both; S1 Table) and did not have a significant lack of memory (MI: p>0.1 for both; S1 Table). In addition, MB310C flies had higher initial levels of courtship (CI_begin_ = 0.39; S1 Table), indicating that the low initial levels at restrictive temperature may be due to the temperature. A recent study identified the PAM α1 neurons and α1 MBONs are required for appetitive olfactory long-term memory [17]. This result is consistent with our hypothesis that courtship memory and appetitive memory circuits overlap anatomically. Taken together, we conclude that α1 MBONs may play a role in courtship conditioning memory.

Other lines expressing in the output neurons that were hits were either the only line expressing in that particular neuron type, making it difficult to make conclusions about these neurons (i.e. MB057B, expressing in β’1 MBONs, S4 Fig), or were expressed in neuron types that generally were not hits, indicating that these neurons likely are not necessary for courtship memory (i.e. MB549C, expressing in α2sc and α’3ap MBONs, S4 and S5 Figs).

### Many PAM Dopaminergic Neurons are Needed for Courtship Memory, but PPL1 Dopaminergic Neurons are not Necessary

Of the eighteen PAM DAN lines tested, ten were memory hits. These lines included PAM neurons projecting into most regions of the MB lobes.

**Fig 4:**
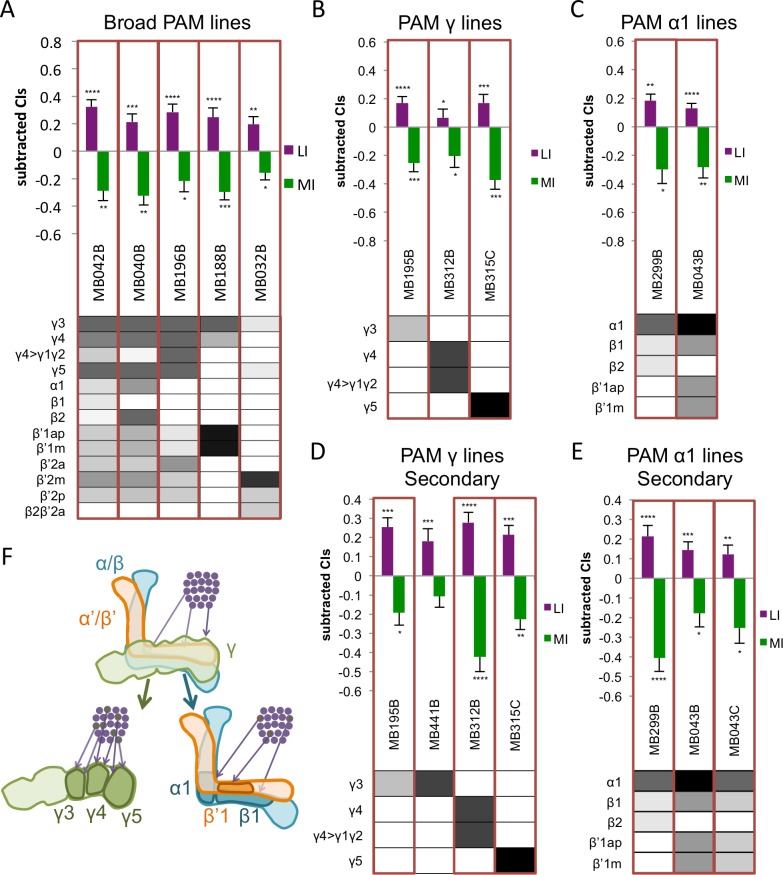
Courtship Conditioning Learning and Memory in PAM Neurons. Learning index (LI) and memory index (MI) for A. broadly-expressed PAM neuron lines, B. PAM γ lines, and C. PAM α1 lines. Lines identified as courtship memory hits are boxed in red. Expression patterns are directly below the LI and MI for each line. Shading indicates relative levels of expression as reported in [35]. Significance is determined using one-sided Wilcoxon signed rank tests with Benjamini-Hochberg post-hoc corrections. *, p < .05; **, p < .01; ***, p < .001; ****, p < .0001. Error bars are SEM, n = 19–24. Secondary screening for D. PAM γ lines and E. PAM α1 lines. Lines identified as courtship memory hits are boxed in red. Expression patterns are directly below the LI and MI for each line. Shading indicates relative levels of expression as reported in [35]. Significance is determined using one-sided Wilcoxon signed rank tests. *, p < .05; **, p < .01; ***, p < .001; ****, p < .0001. Error bars are SEM, n = 17–27. F. Diagram of the mushroom bodies, the general location of the cluster of PAM neuron cell bodies, and the sites of innervation of the γ3, γ5, and α1, β1, β’1 PAM neuron lines [35].

PAM neurons are important for appetitive memory; they are believed to signal reward [5, 13, 15]. In our primary screen, five lines with broad expression in PAM neurons, three of which express in more than five PAM neuron types, were courtship memory hits (Fig 4 and S7 Fig). This result supports the idea that some subset or combination of PAM neurons is important for courtship memory and provides additional support for the overlap of courtship memory and appetitive memory circuits.

Several types of PAM γ neurons are required for courtship memory. One strong memory hit was MB315C, expressing in PAM γ5 neurons (Fig 4 and S7 Fig). These neurons, also called aSP13 neurons, were necessary for courtship memory in other studies [21, 48]. A recent study also found PAM γ5 neurons are necessary for long-term memory in nutrient-dependent appetitive memory [49]. Secondary assays confirmed that PAM γ5 neurons are needed for courtship memory (Fig 4 and S7 Fig). Two other types of PAM γ neurons may also play a role in courtship conditioning memory. Every line expressed in PAM γ3 neurons was a memory hit, including one line specific to these neurons (MB195B, Fig 4 and S7 Fig). In secondary assays, we verified that MB195B was needed for memory, but an additional line expressed in PAM γ3 neurons, MB441B, was not a hit (Fig 4 and S7 Fig). This could be due to differences in expression level in the neurons or differences in genetic background of the lines, as mentioned above. When tested at permissive temperature, MB441B did show significant learning and did not show a significant lack of memory (LI: p<0.05; MI: p>0.3; S1 Table), indicating that the difference between MB195B and MB441B at restrictive temperature is likely due to differences in expression level. Five out of seven lines expressing in PAM γ4 (MB312B (Fig 4), MB188B, MB042B, MB040B, and MB196B (Fig 4)) were hits in the primary screen (Fig 4 and S7 Fig). One line expresses specifically in PAM γ4 and PAM γ4>γ1γ2 neurons, MB312B. We tested this line in secondary screening, and we verified it is a courtship memory hit (Fig 4 and S7 Fig). PAM γ4 neurons have a proposed role in sweet taste reinforcement in appetitive memory [49]; this provides additional support for the neuronal overlap between courtship memory and appetitive memory.

Several lines expressing in the only α lobe region having PAM neuronal projections, α1, lacked courtship memory. These PAM α1 neurons are necessary and sufficient for reward signaling in long-term appetitive olfactory memory [15, 17]. In addition to two broadly expressing lines (MB042B and MB040B, Fig 4), two lines with more specific PAM α1 expression (MB043B and MB299B) were memory hits (Fig 4 and S7 Fig). No lines were available with expression only in PAM α1 neurons; however, MB299B showed only weak expression in two other neuron types (PAM β1 and PAM β2), and MB043B had a high level of expression in PAM α1 (Fig 4). Three other lines expressing in PAM α1, MB047B, MB194B, and MB213B, were not hits, but all of these lines had stronger expression levels in other neurons, indicating that it may be PAM α1 activity that is important for courtship memory (S6 Fig). Because of the hits we identified in the α/β KCs, we tested lines expressing in PAM α1 neurons in secondary assays. Repetition of tests with MB299B and MB043B verified they are each needed for courtship memory, and an additional line with a similar expression pattern to MB043B, MB043C, was also needed for memory (Fig 4 and S7 Fig). However, when tested at permissive temperature, MB043B showed a significant memory (MI: p<0.05; S1 Table) but did not show significant learning (LI: p>0.4; S1 Table), indicating that some background genetic effects may be influencing the MB043B line’s courtship conditioning. Taken together, we suggest that synaptic activity in the PAM α1 neurons is required for courtship memory, but additional experiments will be needed to confirm their role.

We also tested a second cluster of extrinsic dopaminergic neurons in our primary screen, the PPL1 DANs. PPL1 neurons have a known role in aversive, but not appetitive, memory [7, 8, 14, 15]. Of the ten PPL1 lines tested, only one, MB504B, was a memory hit (S6 and S7 Figs). This line expresses in several PPL1 neuron types, and no other line expressing in these neurons was a hit (S6 Fig). These results demonstrate that PPL1 neurons are not needed for courtship memory, just as they are not needed for appetitive memory.

### Other Neuron Types May be Needed for Courtship Memory

We tested six additional lines expressing in other extrinsic mushroom body neurons. We identified hits in two out of three octopaminergic lines and in the MB-C1 neuron (S8 Fig). These hits were not tested in secondary assays.

## Discussion

In this study, we found that at least one type of intrinsic or extrinsic neuron innervating each MB lobe was required for courtship memory.

### Synaptic Signaling in the Mushroom Bodies is not Needed to Learn Courtship Conditioning

The majority of lines tested in the primary screen (67/75) had significant learning. An earlier study found that, even when the mushroom body was completely ablated, learning was not affected [23]. Other types of learning also do not require synaptic signaling in the MBs; both aversive and appetitive memory only require KC activity in retrieval but not acquisition [10, 50]. Because we performed our assays entirely at restrictive temperature, we cannot make conclusions regarding differences between acquisition, consolidation, and retrieval of courtship memory. However, because most of our lines have significant learning, we assume that disruption of storage or retrieval causes our memory hits. Future studies could verify this assumption by inhibiting the function of neurons at different times throughout training and testing.

### Disrupting Synaptic Signaling throughout the Mushroom Bodies does not Affect Courtship Memory

Neither of the two most broadly-expressing KC lines we tested lacked courtship memory. We were surprised by this finding since, based on earlier courtship conditioning studies, we expected that disrupting signaling in the mushroom bodies would cause a lack of memory. However, these studies used methods significantly different from ours: one study used *mud*^*1*^ mutants that lack MBs [24], and the other study completely ablated the MBs by feeding larvae hydroxyurea (HU) [23]. *mud*^*1*^ eliminates the MBs but also affects the central complex and the antennal lobes [51], and HU treatment can affect neurons outside of the MBs [23]. For example, flies with HU-ablated MBs but intact antennal lobes remembered courtship conditioning when tested thirty minutes after training, the time point we tested in our assays [23]. These flies may have courtship memory because other neurons or circuits are compensating for the lack of MBs during development. By contrast, we used shibire^ts^ to inhibit synaptic signaling only in the MBs and only during our assays, allowing for normal development.

One reason our broadly-expressing KC lines may have courtship memory is because the pooled activity of several output neurons may determine the fly’s behavior in response to a stimulus [11, 47]. If this is the case, then a broad disruption of activity may not affect behavior since there is no differential change in neuron responses. For example, if MBON A and MBON B usually both respond with a strength of 1, then if instead they both respond with a strength of 0.1 a downstream neuron pooling these outputs would still see that both neurons responded with the same strength. Only when the activity of subsets of neurons is changed, such as MBON A responding with a strength of 1 and MBON B responding with a strength of 0.1, would the pooled output of the mushroom body also change, leading to a change in behavior.

### Extrinsic γ Neurons, but not γ Kenyon Cells, are needed for Courtship Memory

Different subsets of Kenyon cells are needed for memory in courtship conditioning. The γ lobes seemed to be the best candidate for the site of courtship memory, since this is the location of the majority of fru-positive KCs [39, 40, 52]. While the γ lobes played a vital role in courtship memory in other studies [21, 28, 30, 52], none of the three γ lobe lines we initially tested were hits, and only one out of three γ lobe lines we tested in secondary screening was a hit. This discrepancy could be because the GAL4 lines used in earlier studies are not expressed exclusively in the γ lobes; for example, 201Y, a common γ lobe GAL4 line, also expresses in the α/β lobes and in other brain regions [53]. Additionally, these studies mostly examined changes in courtship memory after knocking down or eliminating a particular gene or protein in the γ lobe (fru^M^, *Orb2*, *DopR1*, and *Hr39* respectively). It is possible that, while the expression of these genes and proteins in the γ lobes is needed for courtship memory, synaptic signaling itself is not needed, as reported in appetitive and aversive olfactory memory [10, 50]. To conclude whether or not synaptic signaling in the γ KCs is needed for courtship memory will require additional experiments, but our results indicate that it is not needed.

Even though the results with the γ KC lines were mostly negative, several γ lobe extrinsic neurons were needed for courtship memory. We confirmed that PAM γ5, a dopaminergic extrinsic neuron, is required for courtship memory, similar to previous results [21]. We also found two other PAM γ neuron types, PAM γ3 and PAM γ4, likely are needed for courtship memory. Output neurons innervating two γ lobe regions, γ3, and γ5, are also required for memory, and output neurons innervating a third region, γ2, may also be required. It is possible that the PAM γ neurons modulate the γ KCs directly and thus form a circuit with the MBONs to generate courtship memory without requiring synaptic signaling. It is also possible that the connections between PAM neurons and MBONs outside of the MBs affect memory formation; for example, PAM γ5 and γ5β’2a MBONs also converge in the superior medial protocerebrum [35]. This could explain why γ KC synaptic activity is not needed while extrinsic γ neurons are needed for courtship memory.

### Courtship Memory and Appetitive Memory Require Similar Subsets of Mushroom Body Neurons

Synaptic signaling in the α/β and in the α’/β’ lobes was needed for courtship memory. Previous studies found that the α/β KCs have roles in courtship conditioning [25, 29, 30]. Blocking activity of α/β neurons by disrupting signaling with tetanus toxin eliminates courtship memory [30]. Expression of DopEcR and OAMB in the α/β lobes is required for courtship memory [25, 29]. In male flies, the α/β lobes, most likely the α/β core (α/βc) neurons, express fru^M^, indicating that they likely play some role in courtship behavior [40, 52]. α/β KCs have been implicated in both aversive and appetitive olfactory memory formation [9]. Specifically, α/βc neurons are important for appetitive memory and α/βs neurons are required for aversive memory [45]. We found that α/β KCs are necessary for courtship memory, and, more specifically, the α/βc neurons are needed. However, our data for the α/βc neurons will need to be confirmed with additional experiments because of inconsistent control data for this line. Our results imply that courtship memory shares common neurons and circuits with appetitive memory.

The requirement of α’/β’ KCs for courtship conditioning memory surprised us. Previous studies have not reported a role for the α’/β’ KCs in learning or memory in courtship conditioning [21, 28, 52]. The α'/β’ lobes are involved in the formation of an early memory trace in aversive memory [54] and in the acquisition and retrieval of short-term aversive and appetitive olfactory memories [9, 44]. It is possible that we have memory hits in the α’/β’ neurons because, by restricting synaptic signaling throughout the training phase, we are disrupting memory storage, retrieval, or both. Since these lines have significant learning, it seems unlikely that memory acquisition is disrupted. Additional experiments shifting the flies to restrictive temperature either during training, during testing, or during the wait between training and testing, instead of throughout the entire assay, could verify whether α’/β’ neurons are necessary for acquisition of courtship memory, for retrieval, or for both.

Many different extrinsic neurons necessary for courtship memory in our assays are also required for one or more types of appetitive memory. PAM DANs in general are important for appetitive memory formation [5, 13, 16] whereas PPL1 DANs are important for aversive memory formation [7, 8, 14, 16]. PAM DANs were required for courtship memory in our assays, but activity of PPL1 neurons was dispensable. Specific subsets of PAM DANs necessary for courtship memory are also important for different types of appetitive memory. PAM α1 may be needed for courtship memory, and these neurons are necessary and sufficient for signaling reward in long-term appetitive olfactory memory [15, 17]. Several PAM γ neurons were necessary for courtship memory, including PAM γ4 and PAM γ5 neurons. A recent study found that PAM γ4 neurons, in combination with PAM β’2am neurons, are necessary for sweet taste reinforcement in appetitive conditioning, and PAM γ5 neurons are necessary for nutrient-dependent long-term appetitive memory [49]. These results are consistent with our hypothesis that courtship memory circuits overlap with appetitive memory circuits.

Several output neuron lines were memory hits in our primary screen, and many of these lines were also hits in multiple appetitive assays using the same MB split-GAL4 lines and a similar shibire^ts^ effector [11]. Lines expressing in γ2α’1 MBONs and α’2 MBONs affected appetitive odor memory and appetitive ethanol memory. The γ5β’2a MBONs and β’2mp MBONs affected appetitive visual memory, appetitive odor memory, and appetitive ethanol memory [11]. Inhibition of each of these types of neurons led to a lack of courtship memory, but this result will need to be confirmed with other assays because of inconsistent control data. Again, these results are consistent with the hypothesis that courtship memory and appetitive memory neuronal circuits overlap.

Based on these results, we propose that courtship memory requires similar neurons and circuits as appetitive memory. This could be because the stimuli involved in courtship conditioning, female flies, are innately of positive valence to a naïve male fly. Initially, the female activates olfactory, visual, and other sensory circuits in a way that indicates she is a desired stimulus. This sensory stimulus may be activating populations of neurons involved in reward processing. Only through training does the male learn to associate those positive sensory cues with a negative stimulus, the lack of successful copulation [21]. A previous study proposed that MBONs do not encode the identity of a stimulus but instead encode the valence of the stimulus [11]. Our results support this idea; several of the MBONs necessary for courtship memory could be responding to a stimulus of positive valence, a female fly, just as MBONS respond to stimuli of positive valence (most often sugars) in appetitive memory. This positive stimulus could become associated with its learned behavioral response, a decrease in courtship, elsewhere in the brain.

While the responses of individual MBONs may be encoding the valence of a stimulus, it has been proposed that the pooling of MBON outputs is what encodes memory. If this is true, then while the MBON types required for courtship memory do overlap with appetitive memory because their stimuli are of positive valence, other output neuron types not needed for appetitive memory may be what distinguishes courtship memory. It could be that it is the pooled activity of all of the required MBONs that encodes courtship memory, and if we were to test the MBONs in combination we would see stronger effects on memory. The MBONs required for appetitive memory are generally glutamatergic or cholinergic while the MBONs needed for aversive memory are GABAergic [11]. The α1 and γ3 MBONs are both GABAergic, and these MBONs currently do not have reported roles in appetitive or aversive memory [11]; they could be needed to encode the negative association between the female trainer and the lack of copulation in courtship memory. The pooling of output from these GABAergic MBONs with output from MBONs involved in appetitive memory assays could form the representation of courtship memory, pairing positive stimuli from multiple sensory modalities with a negative reinforcement, the lack of successful copulation.

These results lay the groundwork for future courtship conditioning studies, narrowing down the mushroom body regions and neuron types of particular interest. Additionally, we found strong overlap between neurons required for courtship memory and neurons required for appetitive memory. Future studies may discover more insights into the nature of memory by further investigating the similarities and differences between these types of memory.

## Materials and Methods

### Fly Lines

Flies were raised on standard molasses media. Flies used in assays were collected 1–4 hours after eclosion and aged 4–6 days at 18C and 50% relative humidity with a 12 hour light/dark cycle before the start of experiments. Females were held in groups ≤15, and males were held individually until used.

Dickinson lab wildtype (DL) was used as a control because it showed the best learning and memory of three wild-types examined (S1 Fig). All females were DL. Don juan::GFP was described previously [55]. MB split-GAL4 lines were described previously [35]. pJFRC100-20xUAS-TTS-Shibire^ts1^-p10 (VK0005) [56] was outcrossed into DL for ten generations prior to use.

### Overview of Courtship Conditioning Assays

Males were tested for learning and memory with respect to courtship conditioning similar to what was done in previous studies [19, 28, 29, 38]. Briefly, a mated female (the trainer, prepared as described below) was loaded into the lower half of a courtship chamber, and the male fly was loaded into the upper half. The flies were allowed to acclimatize at the desired temperature in the divided courtship chamber for approximately 1 hour and were kept at this temperature throughout the entire assay. At the start of the assay the barrier dividing the chamber was removed, and the male fly was permitted to court the trainer during a one-hour training period that was recorded. The male was then transferred to a new chamber with a decapitated virgin female (the tester, prepared as described below) preloaded in the lower half. After a thirty-minute wait, the barrier between the upper and the lower halves was removed, and the male could court the tester for ten minutes, which was also recorded. DL wild-type flies were tested at various points throughout the screen, and heterozygous shibire effector flies (crossed with DL wild-type) exhibited both learning and memory in our courtship conditioning assay (p<0.01 for LIs, p>0.2 for MIs, assays conducted at both 25C and 32C).

### Courtship Conditioning Assay Parameters

Courtship chambers were cylindrical in shape and consisted of two acrylic trays stacked on top of one another. Each had openings with a diameter of 8mm and a height of 3mm such that when they were stacked they formed chambers 8mm in diameter and 6mm in height. Males and females were kept separated by a transparent plastic divider placed between the top and bottom trays. All flies were loaded and transferred by aspiration. Restrictive temperature was 32C and permissive temperature was 22C or 25C.

### Generation of Mated Female Trainers

Mated female trainers were generated 18–24 hours before use by one of two procedures. Initially, females were mated by pairing one virgin female with one or two DL males for 1–2 hours in a small vial with fly food. Pairs that mated were marked, and these females were removed after copulation and grouped in a fresh food vial. Because of the time needed to observe the flies mating, we adopted another, more efficient method to generate mated females. A group of virgin females was paired with a group of Don juan::GFP (Dj::GFP) males for approximately two hours in a bottle with fly food. Dj::GFP males have a sperm specific protein component tagged with GFP [55]. Males and females were separated, then females were sorted by whether their abdomens were fluorescent, meaning they had Dj::GFP sperm and thus had mated. Mated females were grouped in fresh food vials. DL controls demonstrated learning and memory when trained with Dj::GFP-mated females (learning index = 0.13±0.04, p = 0.0004; memory index = -0.16±0.09, p = 0.09).

### Generation of Decapitated Female Testers

In order to assay generalized courtship memory, meaning training with one type of female (mated) and testing with another type (virgin) [22], we used decapitated virgin females as testers. Assaying generalized courtship conditioning means we are testing more broadly for memory towards females rather than simply testing the ability of the fly to form an association between specific cues (like cis-vaccenyl acetate) and reduction of courtship. Virgin females under cold anesthesia were decapitated with a scalpel approximately one hour before the test period. Testers were allowed to recover for a few minutes, then testers that had regained upright posture or that had wing or leg movements were loaded into new courtship chambers.Secondary Experiments

Lines of interest were tested in secondary experiments. These experiments were performed exactly the same as the primary screen experiments, the only difference being that they consisted of a new genetic replicate. Each split-GAL4 line was crossed a second time with the shibire^ts^ effector. Males were collected, held for 4–6 days, then tested in our courtship conditioning paradigm as described above.

Permissive temperature controls were performed similarly to secondary restrictive temperature experiments. Flies consisted of a new genetic replicate with each split-GAL4 line crossed with the shibirets effector. Males were collected, held for 4–6 days, then tested at permissive temperature in our courtship conditioning paradigm as described above. Testing a new genetic replicate at permissive temperature allowed us to better examine background genetic effects in our screen, compared to testing a split-GAL4 line without first crossing it to the shibire^ts^ effector.

### Video Recording Hardware and Software

The 60 minute training period and the 10 minute test period were recorded using Hitachi KP-M2AN, StingRay F125C, or Canon Vixia HF R500 cameras. Hitachi and StingRay movies were recorded using either iMovie or Quicktime Pro 7 software.

### Data Analysis

Videos were analyzed by hand using LifeSong X software (http://lifesong.bio.brandeis.edu/). For each fly, three different courtship indices (CIs), the amount of time the male courted, were calculated: first, the CI during the first ten minutes of training (CI_begin_), second, the CI during the last ten minutes of training (CI_end_), and third, the CI during the ten minute test (CI_test_). Male flies that did not court during the first ten minutes of training (CI_begin_ = 0) and male flies that copulated during training were excluded from analysis.

In our assays, we kept track of individual males so that we could follow changes in the behavior of individual flies throughout training and testing. This is different from previous studies, which did not calculate CIs during the training but instead tested learning or memory by comparing CI_test_ for trained flies versus naïve flies on a population level [21, 28, 30, 57]. By calculating CIs during the training period and following individual males from training to testing, we can observe learning and memory on a per fly basis.

Because of this added rigor, we calculate both learning and memory indices for each fly. We calculate a learning index as LI = CI_begin_−CI_end_ and a memory index as MI = CI_end_−CI_test_. These values are then averaged together across all flies for each genotype. In summary, we generate LIs and MIs by subtracting individual flies’ CIs then averaging those subtractions, rather than averaging individuals’ CIs then subtracting those averages.

We chose our learning and memory indices so that we can calculate significance using one-sided comparisons. If learning occurred, CI_end_ will be significantly less than CI_begin_ (LI>0). Instead of comparing CI_begin_ with CI_test_ to determine if memory occurred, we compare CI_end_ with CI_test_. By comparing CI_end_ with CI_test_, we assume that if memory occurred then these two values will not be significantly different (MI = 0). If no memory occurred, then CI_test_ will be significantly greater than CI_end_ (MI<0). Thus, our learning index is one-sided to allow testing for values significantly above zero, and our memory index is one-sided to allow testing for values significantly below zero. To calculate statistical significance, we used Wilcoxon signed ranks tests with a Benjamini-Hochberg post-hoc correction for multiple comparisons on the primary screen data. Excel and custom Matlab scripts were used to analyze data.

## Supporting Information

S1 Fig Courtship Conditioning in Wild-type Lines. A. Three wild-type lines, CSA, CSMH, and DL, were tested in courtship conditioning assays. DL had the strongest levels of courtship learning and memory. Significance is determined using one-sided Wilcoxon signed rank tests. *, p < .05; **, p < .01; ***, p < .001; ****, p < .0001. Error bars are SEM, n = 13–48. B. Learning index (LI) and memory index (MI) for wild-type lines. A significant LI indicates learning occurred, and a significant MI indicates a lack of memory (conversely, a not significant MI indicates memory occurred). Significance is determined using one-sided Wilcoxon signed rank tests. *, p < .05; **, p < .01; ***, p < .001; ****, p < .0001. Error bars are SEM, n = 13–48.
(PPTX)

S2 Fig Learning and Memory in Additional Kenyon Cell Lines. A. Learning index (LI) and memory index (MI) for γ Kenyon cell lines. Lines identified as courtship memory hits are boxed in red. Expression patterns are directly below the LI and MI for each line. Shading indicates relative levels of expression in each neuron type as reported in (35). Significance is determined using one-sided Wilcoxon signed rank tests with Benjamini-Hochberg post-hoc corrections. *, p < .05; **, p < .01; ***, p < .001; ****, p < .0001. Error bars are SEM, n = 20–22. B. Secondary screening for γ KC lines. Only one out of three lines, MB419B, was a memory hit. Expression patterns are directly below the LI and MI for each line. Shading indicates relative levels of expression of each neuron type as reported in (35). Significance is determined using one-sided Wilcoxon signed rank tests. *, p < .05; **, p < .01; ***, p < .001; ****, p < .0001. Error bars are SEM, n = 15–21. C. Secondary screening for α’/β’ KC lines. These lines were not memory hits because of low initial courtship levels. Expression patterns are directly below the LI and MI for each line. Shading indicates relative levels of expression of each neuron type as reported in (35). Significance is determined using one-sided Wilcoxon signed rank tests. *, p < .05; **, p < .01; ***, p < .001; ****, p < .0001. Error bars are SEM, n = 17, 14. D. Learning and memory in broad Kenyon cell lines. Lines identified as courtship memory hits are boxed in red. Expression patterns are directly below the LI and MI for each line. Shading indicates relative levels of expression in each neuron type as reported in (35). Significance is determined using one-sided Wilcoxon signed rank tests with Benjamini-Hochberg post-hoc corrections. *, p < .05; **, p < .01; ***, p < .001; ****, p < .0001. Error bars are SEM, n = 20–24.
(PPTX)

S3 Fig Courtship Indices of Kenyon Cell Lines. A. Courtship indices for the three periods observed, CI_begin_, CI_end_, and CI_test_, for KC lines tested in primary screening. Significance is determined using one-sided Wilcoxon signed rank tests with Benjamini-Hochberg post-hoc corrections. *, p < .05; **, p < .01; ***, p < .001; ****, p < .0001. Error bars are SEM, n = 19–24. B. Courtship indices for the three periods observed, CI_begin_, CI_end_, and CI_test_, for KC lines tested in secondary screening. Significance is determined using one-sided Wilcoxon signed rank tests. *, p < .05; **, p < .01; ***, p < .001; ****, p < .0001. Error bars are SEM, n = 14–46.
(PPTX)

S4 Fig Learning and Memory in Additional Output Neuron Lines. A. Learning index (LI) and memory index (MI) for A. α2 and α3 MBON lines, B. β2β’2a lines, C. other MBON hits, and D. other MBON lines. Lines identified as courtship memory hits are boxed in red. Expression patterns are directly below the LI and MI for each line. Shading indicates relative levels of expression in each neuron type as reported in (35). Significance is determined using one-sided Wilcoxon signed rank tests with Benjamini-Hochberg post-hoc corrections. *, p < .05; **, p < .01; ***, p < .001; ****, p < .0001. Error bars are SEM, n = 10–23.
(PPTX)

S5 Fig Courtship Indices in MB Output Neurons. A. Courtship indices for the three periods observed, CI_begin_, CI_end_, and CI_test_, for MBON lines tested in primary screening. Significance is determined using one-sided Wilcoxon signed rank tests with Benjamini-Hochberg post-hoc corrections. *, p < .05; **, p < .01; ***, p < .001; ****, p < .0001. Error bars are SEM, n = 9–24. B. Courtship indices for the three periods observed, CI_begin_, CI_end_, and CI_test_, for MBON lines tested in secondary screening. Significance is determined using one-sided Wilcoxon signed rank tests. *, p < .05; **, p < .01; ***, p < .001; ****, p < .0001. Error bars are SEM, n = 8–46.
(PPTX)

S6 Fig Learning and Memory in Additional Dopaminergic Neuron Lines. A. Learning index (LI) and memory index (MI) for PAM DAN lines. Expression patterns are directly below the LI and MI for each line. Shading indicates relative levels of expression in each neuron type as reported in (35). Significance is determined using one-sided Wilcoxon signed rank tests with Benjamini-Hochberg post-hoc corrections. *, p < .05; **, p < .01; ***, p < .001; ****, p < .0001. Error bars are SEM, n = 15–24. B. Learning and memory in PPL1 DAN lines. The line identified as a courtship memory hit is boxed in red. Expression patterns are directly below the LI and MI for each line. Shading indicates relative levels of expression in each neuron type as reported in (35). Significance is determined using one-sided Wilcoxon signed rank tests with Benjamini-Hochberg post-hoc corrections. *, p < .05; **, p < .01; ***, p < .001; ****, p < .0001. Error bars are SEM, n = 17–24.
(PPTX)

S7 Fig Courtship Indices in DAN Neurons. A. Courtship indices for the three periods observed, CI_begin_, CI_end_, and CI_test_, for PAM lines tested in primary screening. Significance is determined using one-sided Wilcoxon signed rank tests with Benjamini-Hochberg post-hoc corrections. *, p < .05; **, p < .01; ***, p < .001; ****, p < .0001. Error bars are SEM, n = 15–24. B. Courtship indices for the three periods observed, CI_begin_, CI_end_, and CI_test_, for PAM lines tested in secondary screening. Significance is determined using one-sided Wilcoxon signed rank tests. *, p < .05; **, p < .01; ***, p < .001; ****, p < .0001. Error bars are SEM, n = 17–27. C. Courtship indices for the three periods observed, CI_begin_, CI_end_, and CI_test_, for PPL1 lines tested in primary screening. Significance is determined using one-sided Wilcoxon signed rank tests with Benjamini-Hochberg post-hoc corrections. *, p < .05; **, p < .01; ***, p < .001; ****, p < .0001. Error bars are SEM, n = 17–24.
(PPTX)

S8 Fig Learning and Memory in Other Extrinsic MB Neurons. A. Learning index (LI) and memory index (MI) for octopaminergic neuron lines and other extrinsic MB lines. Lines identified as courtship memory hits are boxed in red. Expression patterns are directly below the LI and MI for each line. Shading indicates relative levels of expression as reported in (35). Significance is determined using one-sided Wilcoxon signed rank tests with Benjamini-Hochberg post-hoc corrections. *, p < .05; **, p < .01; ***, p < .001; ****, p < .0001. Error bars are SEM, n = 11–22. B. Courtship indices for the three periods observed, CI_begin_, CI_end_, and CI_test_, for octopaminergic lines and other MB extrinsic neuron lines tested in secondary screening. Significance is determined using one-sided Wilcoxon signed rank tests with Benjamini-Hochberg post-hoc corrections. *, p < .05; **, p < .01; ***, p < .001; ****, p < .0001. Error bars are SEM, n = 11–22.
(PPTX)

S1 Table Learning Indices, Memory Indices, and Courtship Indices. All data included here is for flies of the given split-GAL4 line crossed with a 20xUAS-shibire^ts^ effector and tested at 32C in our courtship conditioning paradigm. The first worksheet lists the data for the primary screen, and the second worksheet lists the data for the secondary assays. In each worksheet, the first two columns list the cell type and the split-GAL4 line name. The next columns are the average learning and memory indices and the standard errors for the learning and memory indices. The one-sided p values for the learning and memory indices are listed next. Significance was calculated using Wilcoxon signed ranks with a Benjamini-Hochberg correction for multiple comparisons used on the primary screen data. After those are the average courtship indices for each of the three periods analyzed, beginning of training, end of training, and test, and the standard errors for each of those. Last, the n for each genotype is listed. KC, Kenyon Cell; MBON, mushroom body output neuron; PPL1, protocerebral posterior lateral 1; PAM, protocerebral anterior medial; OA, octopaminergic; CSD, serotonergic neuron; MB-C1, GABAergic neuron; SIFamide, peptidergic neuron. See (35) for description of neuron types.
(XLSX)
