# Supplementary material for: Memory Elicited by Courtship Conditioning Requires Mushroom Body Neuronal Subsets Similar to Those Utilized in Appetitive Memory
Source: PLoS One. 2016 Oct 20;11(10):e0164516. doi: 10.1371/journal.pone.0164516 (PMC5072562; doi:10.1371/journal.pone.0164516)
Supplement: S1 Fig — A. Three wild-type lines, CSA, CSMH, and DL, were tested in courtship conditioning assays. DL had the strongest levels of courtship learning and memory. Significance is determined using one-sided Wilcoxon signed rank tests. *, p < .05; **, p < .01; ***, p < .001; ****, p < .0001. Error bars are SEM, n = 13–48. B. Learning index (LI) and memory index (MI) for wild-type lines. A significant LI indicates learning occurred, and a significant MI indicates a lack of memory (conversely, a not significant MI indicates memory occurred). Significance is determined using one-sided Wilcoxon signed rank tests. *, p < .05; **, p < .01; ***, p < .001; ****, p < .0001. Error bars are SEM, n = 13–48. (PPTX) [file pone.0164516.s001.pptx]

## Slide 1
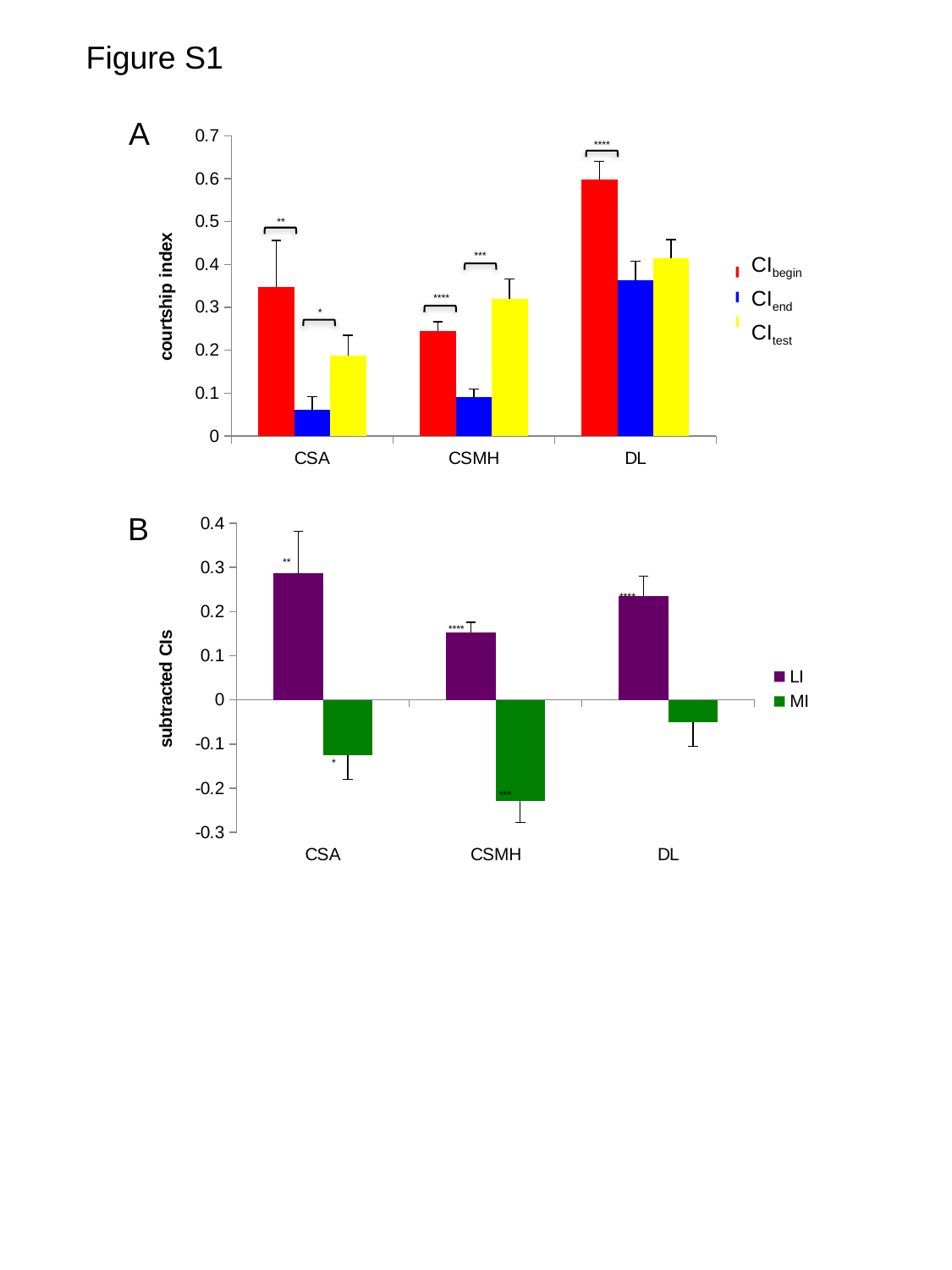

Figure S1
A
### Chart
| Category | CIbegin | CIend | CItest |
|---|---|---|---|
| CSA | 0.346518333333333 | 0.0600919230769231 | 0.186553974358974 |
| CSMH | 0.24381880952381 | 0.090639387755102 | 0.319019081632653 |
| DL | 0.598351393034826 | 0.36325447761194 | 0.41447855721393 |****
**
***
CIbegin
CIend
****
*
CItest
B
### Chart
| Category | LI | MI |
|---|---|---|
| CSA | 0.28642641025641 | -0.126462051282051 |
| CSMH | 0.153179421768707 | -0.228379693877551 |
| DL | 0.235096915422886 | -0.0512240796019901 |**
****
****
*
***
