# Supplementary material for: Memory Elicited by Courtship Conditioning Requires Mushroom Body Neuronal Subsets Similar to Those Utilized in Appetitive Memory
Source: PLoS One. 2016 Oct 20;11(10):e0164516. doi: 10.1371/journal.pone.0164516 (PMC5072562; doi:10.1371/journal.pone.0164516)
Supplement: S2 Fig — A. Learning index (LI) and memory index (MI) for γ Kenyon cell lines. Lines identified as courtship memory hits are boxed in red. Expression patterns are directly below the LI and MI for each line. Shading indicates relative levels of expression in each neuron type as reported in (35). Significance is determined using one-sided Wilcoxon signed rank tests with Benjamini-Hochberg post-hoc corrections. *, p < .05; **, p < .01; ***, p < .001; ****, p < .0001. Error bars are SEM, n = 20–22. B. Secondary screening for γ KC lines. Only one out of three lines, MB419B, was a memory hit. Expression patterns are directly below the LI and MI for each line. Shading indicates relative levels of expression of each neuron type as reported in (35). Significance is determined using one-sided Wilcoxon signed rank tests. *, p < .05; **, p < .01; ***, p < .001; ****, p < .0001. Error bars are SEM, n = 15–21. C. Secondary screening for α’/β’ KC lines. These lines were not memory hits because of low initial courtship levels. Expression patterns are directly below the LI and MI for each line. Shading indicates relative levels of expression of each neuron type as reported in (35). Significance is determined using one-sided Wilcoxon signed rank tests. *, p < .05; **, p < .01; ***, p < .001; ****, p < .0001. Error bars are SEM, n = 17, 14. D. Learning and memory in broad Kenyon cell lines. Lines identified as courtship memory hits are boxed in red. Expression patterns are directly below the LI and MI for each line. Shading indicates relative levels of expression in each neuron type as reported in (35). Significance is determined using one-sided Wilcoxon signed rank tests with Benjamini-Hochberg post-hoc corrections. *, p < .05; **, p < .01; ***, p < .001; ****, p < .0001. Error bars are SEM, n = 20–24. (PPTX) [file pone.0164516.s002.pptx]

## Slide 1
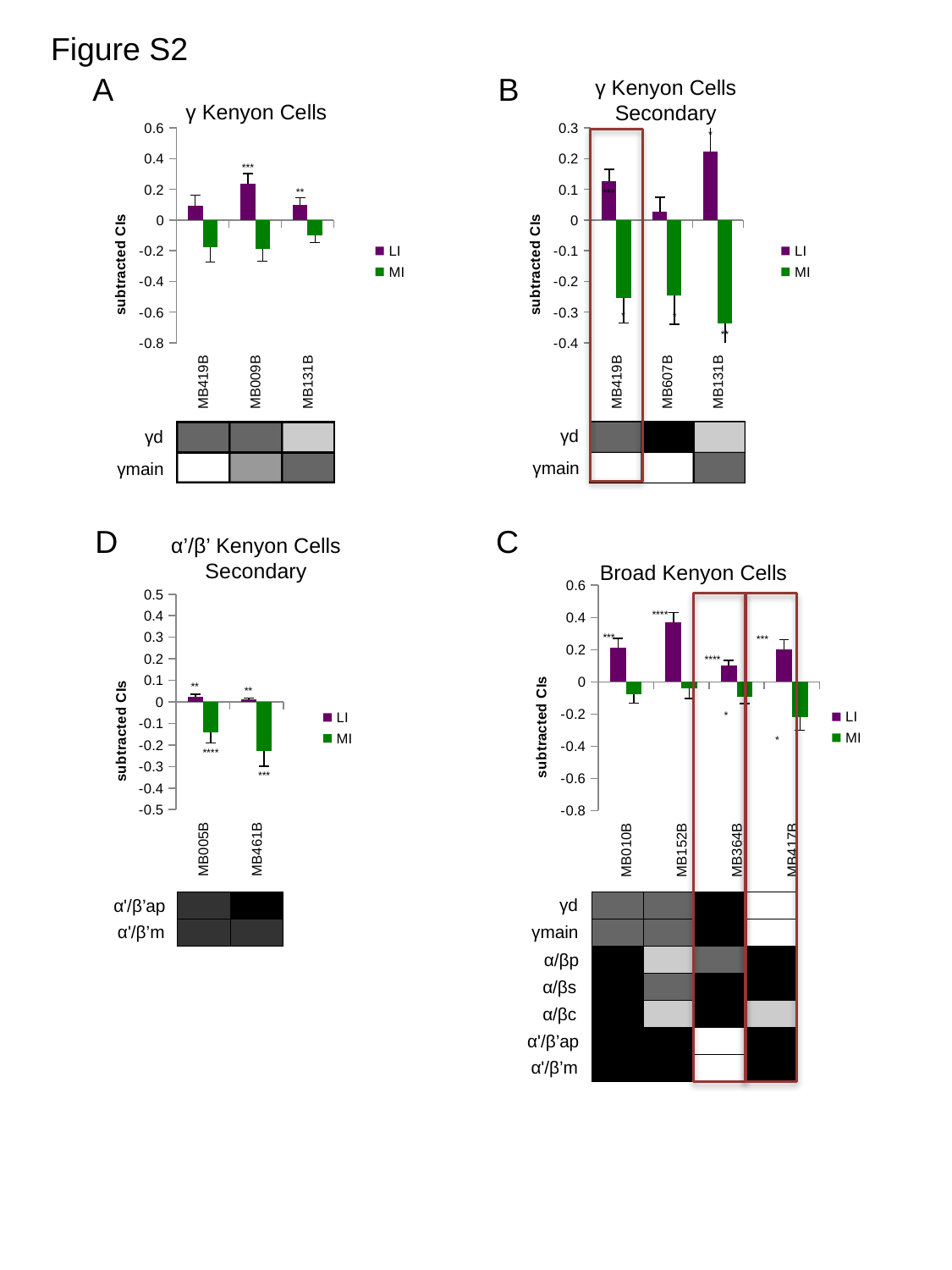

Figure S2
A
B
γ Kenyon Cells
Secondary
γ Kenyon Cells
### Chart
| Category | | |
|---|---|---|
| MB419B | 0.0929782575757575 | -0.178811666666667 |
| MB009B | 0.236254833333333 | -0.185971333333333 |
| MB131B | 0.0991860606060606 | -0.0998754545454546 |
### Chart
| Category | LI | MI |
|---|---|---|
| MB419B | 0.127717777777778 | -0.2548874075 |
| MB607B | 0.0285596825714286 | -0.246754126952381 |
| MB131B | 0.224476 | -0.336434 |*
***
**
***
*
*
**
γd
γd
γmain
γmain
C
D
α’/β’ Kenyon Cells
Secondary
Broad Kenyon Cells
### Chart
| Category | | |
|---|---|---|
| MB010B | 0.212228174603175 | -0.0759979365079365 |
| MB152B | 0.370140277777778 | -0.0417334027777778 |
| MB364B | 0.101931875 | -0.0925014583333334 |
| MB417B | 0.203761333333333 | -0.219237 |
### Chart
| Category | | |
|---|---|---|
| MB005B | 0.0233425490196078 | -0.141172941176471 |
| MB461B | 0.01358 | -0.228551904761905 |
****
***
***
****
**
**
*
*
****
***
γd
α'/β’ap
| | | | |
| --- | --- | --- | --- |
| | | | |
| | | | |
| | | | |
| | | | |
| | | | |
| | | | |
| | |
| --- | --- |
| | |
γmain
α'/β’m
α/βp
α/βs
α/βc
α'/β’ap
α'/β’m
