# Supplementary material for: Memory Elicited by Courtship Conditioning Requires Mushroom Body Neuronal Subsets Similar to Those Utilized in Appetitive Memory
Source: PLoS One. 2016 Oct 20;11(10):e0164516. doi: 10.1371/journal.pone.0164516 (PMC5072562; doi:10.1371/journal.pone.0164516)
Supplement: S3 Fig — A. Courtship indices for the three periods observed, CIbegin, CIend, and CItest, for KC lines tested in primary screening. Significance is determined using one-sided Wilcoxon signed rank tests with Benjamini-Hochberg post-hoc corrections. *, p < .05; **, p < .01; ***, p < .001; ****, p < .0001. Error bars are SEM, n = 19–24. B. Courtship indices for the three periods observed, CIbegin, CIend, and CItest, for KC lines tested in secondary screening. Significance is determined using one-sided Wilcoxon signed rank tests. *, p < .05; **, p < .01; ***, p < .001; ****, p < .0001. Error bars are SEM, n = 14–46. (PPTX) [file pone.0164516.s003.pptx]

## Slide 1
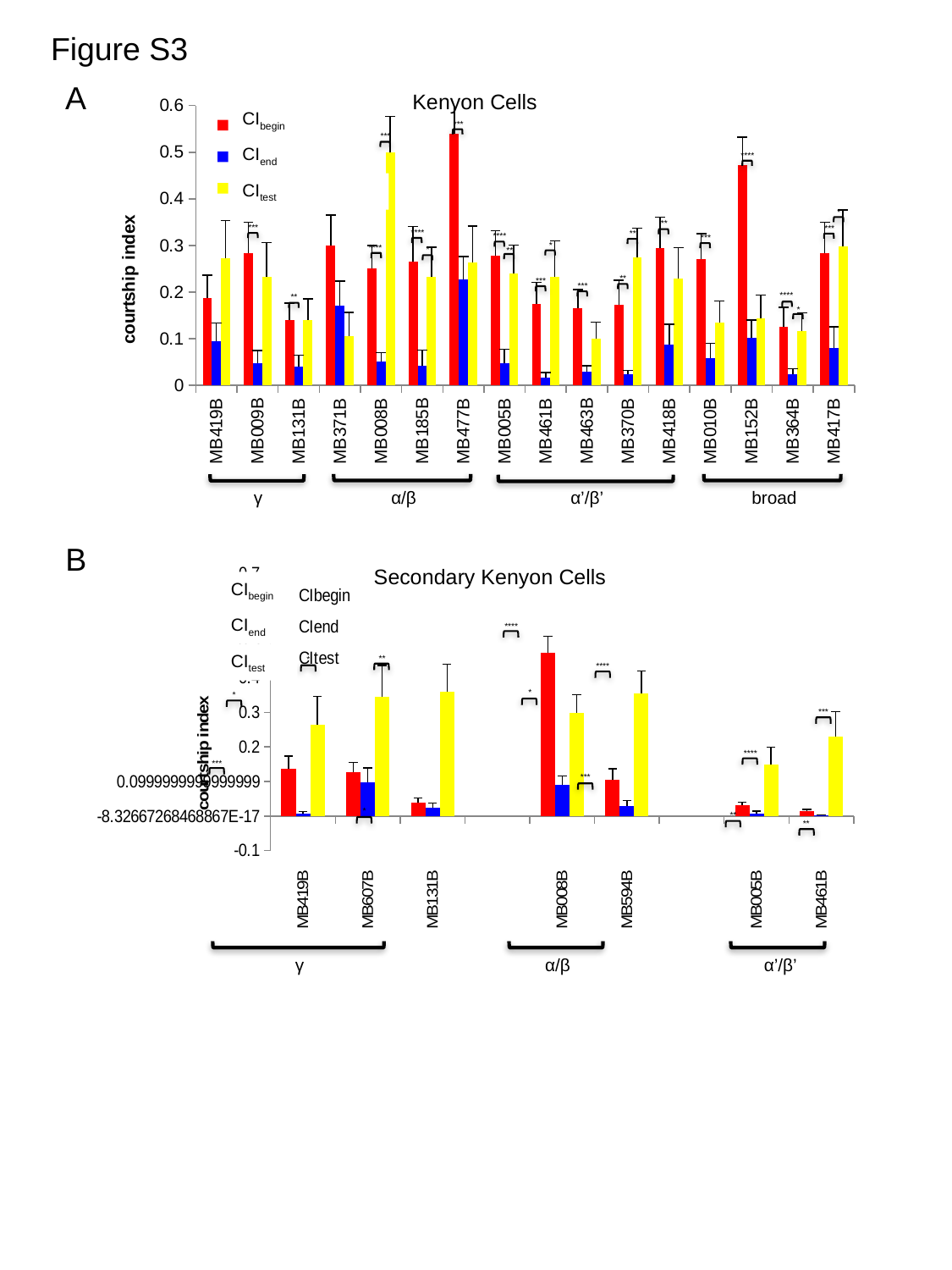

Figure S3
A
Kenyon Cells
### Chart
| Category | | | |
|---|---|---|---|
| MB419B | 0.187269015 | 0.094290758 | 0.273102424 |
| MB009B | 0.283524833333333 | 0.04727 | 0.233241333333333 |
| MB131B | 0.139953712121212 | 0.0407676515151515 | 0.140643106060606 |
| MB371B | 0.299699210526316 | 0.171301403508772 | 0.105902456140351 |
| MB008B | 0.250168125 | 0.0505366666666667 | 0.499141458333333 |
| MB185B | 0.26622253968254 | 0.0420296031746032 | 0.232518253968254 |
| MB477B | 0.540470208333333 | 0.227020972222222 | 0.263861666666667 |
| MB005B | 0.278301166666667 | 0.0464451666666667 | 0.240460333333333 |
| MB461B | 0.175094649122807 | 0.0168932456140351 | 0.232904298245614 |
| MB463B | 0.164604930555556 | 0.0292729861111111 | 0.0993749305555555 |
| MB370B | 0.172676449275362 | 0.0240026811594203 | 0.27472652173913 |
| MB418B | 0.293956269825397 | 0.0867970634920635 | 0.228041587301587 |
| MB010B | 0.269930317460317 | 0.0577021428571428 | 0.133700079365079 |
| MB152B | 0.472346666666667 | 0.102206388888889 | 0.143939791666667 |
| MB364B | 0.124980486111111 | 0.0230486111111111 | 0.115550069444444 |
| MB417B | 0.283142166666667 | 0.0793808333333333 | 0.298617833333333 |CIbegin
***
***
CIend
****
CItest
*
**
***
***
****
**
****
***
*
****
*
**
**
***
***
****
**
*
α/β
α’/β’
broad
γ
B
### Chart
| Category | | | |
|---|---|---|---|
| MB419B | 0.135799722222222 | 0.00808194444444444 | 0.262969351851852 |
| MB607B | 0.126613412698413 | 0.0980537301587301 | 0.344807857142857 |
| MB131B | 0.0379301111111111 | 0.0234661111111111 | 0.359900111111111 |
| | None | None | None |
| MB008B | 0.47155768115942 | 0.0899240942028985 | 0.298588804347826 |
| MB594B | 0.103782933333333 | 0.0294458 | 0.354394733333333 |
| | None | None | None |
| MB005B | 0.0313600980392157 | 0.00801754901960784 | 0.149190490196078 |
| MB461B | 0.0153260714285714 | 0.00174607142857143 | 0.230297976190476 |****
**
*
****
*
*
***
****
***
***
*
**
**
Secondary Kenyon Cells
CIbegin
CIend
CItest
γ
α/β
α’/β’
