# Supplementary material for: Memory Elicited by Courtship Conditioning Requires Mushroom Body Neuronal Subsets Similar to Those Utilized in Appetitive Memory
Source: PLoS One. 2016 Oct 20;11(10):e0164516. doi: 10.1371/journal.pone.0164516 (PMC5072562; doi:10.1371/journal.pone.0164516)
Supplement: S4 Fig — A. Learning index (LI) and memory index (MI) for A. α2 and α3 MBON lines, B. β2β’2a lines, C. other MBON hits, and D. other MBON lines. Lines identified as courtship memory hits are boxed in red. Expression patterns are directly below the LI and MI for each line. Shading indicates relative levels of expression in each neuron type as reported in (35). Significance is determined using one-sided Wilcoxon signed rank tests with Benjamini-Hochberg post-hoc corrections. *, p < .05; **, p < .01; ***, p < .001; ****, p < .0001. Error bars are SEM, n = 10–23. (PPTX) [file pone.0164516.s004.pptx]

## Slide 1
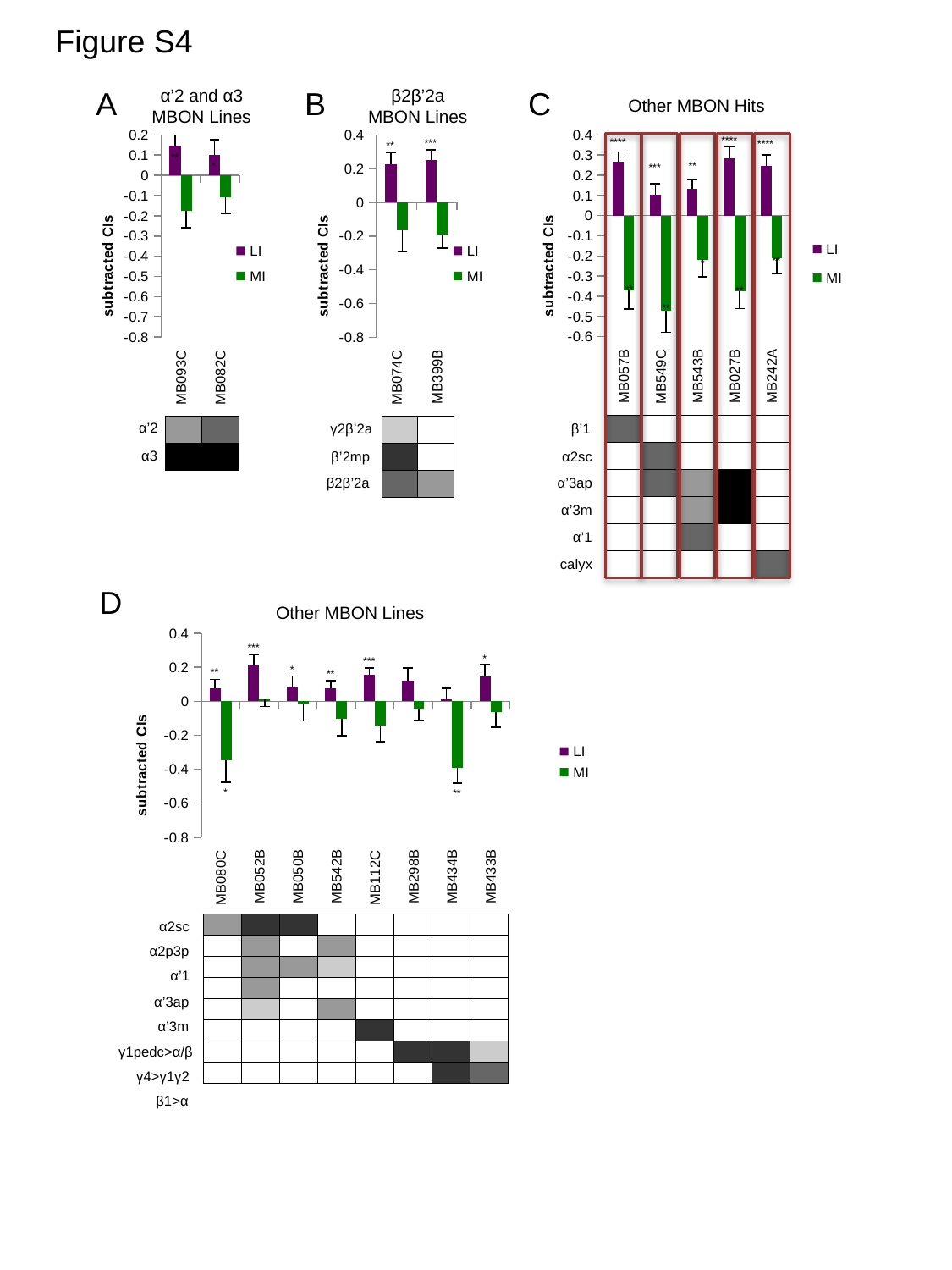

Figure S4
α’2 and α3
MBON Lines
B
β2β’2a
MBON Lines
C
A
Other MBON Hits
### Chart
| Category | LI | MI |
|---|---|---|
| MB093C | 0.148691296296296 | -0.173489444444444 |
| MB082C | 0.10149 | -0.110519210526316 |
### Chart
| Category | LI | MI |
|---|---|---|
| MB074C | 0.226104803921569 | -0.167659705882353 |
| MB399B | 0.250154015151515 | -0.190444318181818 |
### Chart
| Category | LI | MI |
|---|---|---|
| MB057B | 0.266024924242424 | -0.369429393939394 |
| MB549C | 0.103510555555556 | -0.474096222222222 |
| MB543B | 0.130857348484849 | -0.218939090909091 |
| MB027B | 0.282937727272727 | -0.374781363636364 |
| MB242A | 0.244396041666667 | -0.210179444444444 |****
****
***
****
**
**
**
*
***
**
*
**
**
**
α’2
γ2β’2a
β’1
| | | | | |
| --- | --- | --- | --- | --- |
| | | | | |
| | | | | |
| | | | | |
| | | | | |
| | | | | |
| | |
| --- | --- |
| | |
| | |
| --- | --- |
| | |
| | |
α3
α2sc
β’2mp
α’3ap
β2β’2a
α’3m
α’1
calyx
D
Other MBON Lines
### Chart
| Category | LI | MI |
|---|---|---|
| MB080C | 0.0761408333333333 | -0.3490365 |
| MB052B | 0.2146545 | 0.0150335 |
| MB050B | 0.0843367948717949 | -0.0118710256410256 |
| MB542B | 0.0754984848484848 | -0.105027121212121 |
| MB112C | 0.155139895833333 | -0.1423759375 |
| MB298B | 0.121183529411765 | -0.0410678431372549 |
| MB434B | 0.0175221739130435 | -0.39422731884058 |
| MB433B | 0.14782 | -0.0612688095238095 |***
*
***
*
**
**
*
**
α2sc
| | | | | | | | |
| --- | --- | --- | --- | --- | --- | --- | --- |
| | | | | | | | |
| | | | | | | | |
| | | | | | | | |
| | | | | | | | |
| | | | | | | | |
| | | | | | | | |
| | | | | | | | |
α2p3p
α’1
α’3ap
α’3m
γ1pedc>α/β
γ4>γ1γ2
β1>α
