# Supplementary material for: Memory Elicited by Courtship Conditioning Requires Mushroom Body Neuronal Subsets Similar to Those Utilized in Appetitive Memory
Source: PLoS One. 2016 Oct 20;11(10):e0164516. doi: 10.1371/journal.pone.0164516 (PMC5072562; doi:10.1371/journal.pone.0164516)
Supplement: S5 Fig — A. Courtship indices for the three periods observed, CIbegin, CIend, and CItest, for MBON lines tested in primary screening. Significance is determined using one-sided Wilcoxon signed rank tests with Benjamini-Hochberg post-hoc corrections. *, p < .05; **, p < .01; ***, p < .001; ****, p < .0001. Error bars are SEM, n = 9–24. B. Courtship indices for the three periods observed, CIbegin, CIend, and CItest, for MBON lines tested in secondary screening. Significance is determined using one-sided Wilcoxon signed rank tests. *, p < .05; **, p < .01; ***, p < .001; ****, p < .0001. Error bars are SEM, n = 8–46. (PPTX) [file pone.0164516.s005.pptx]

## Slide 1
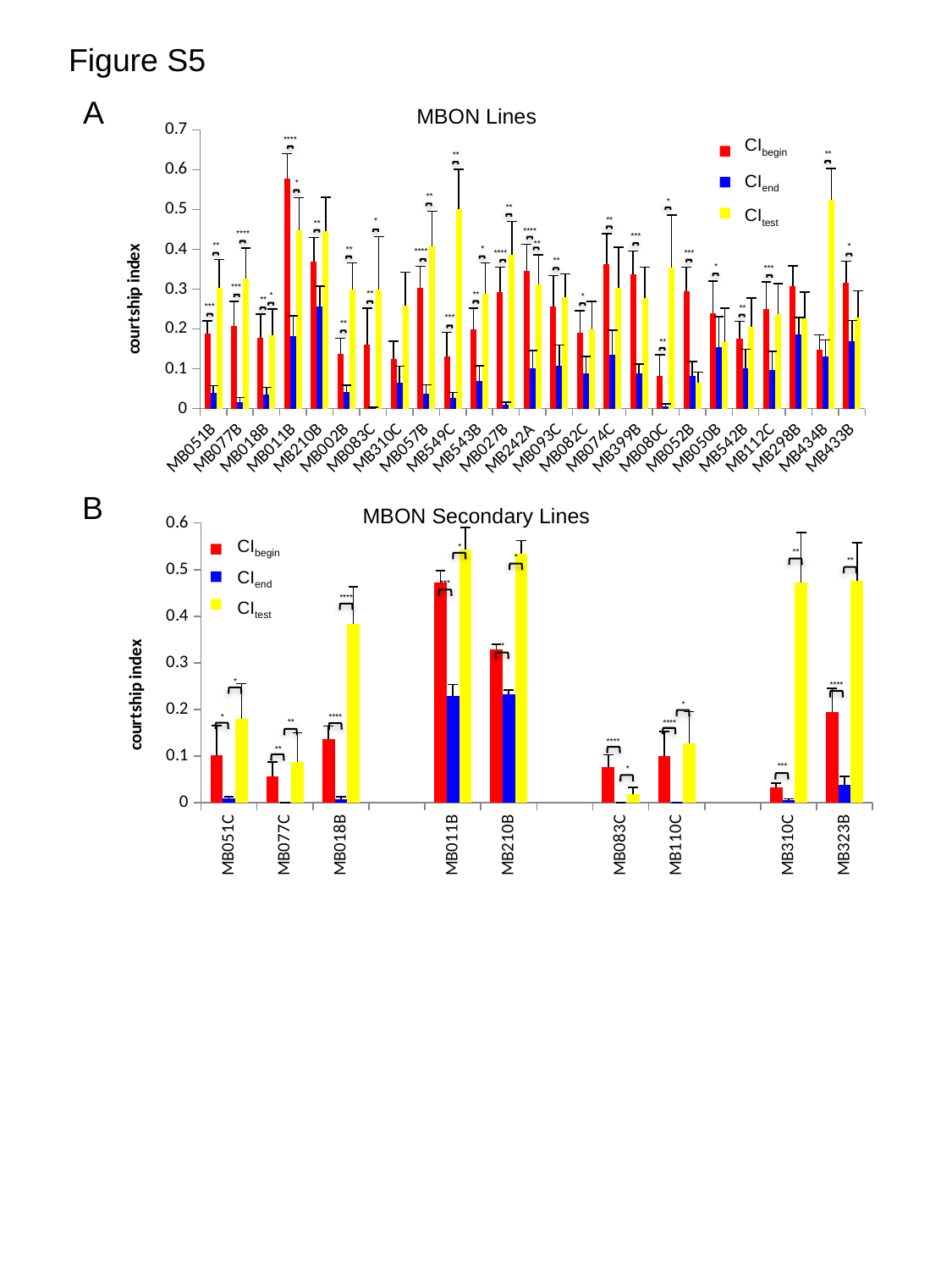

Figure S5
A
MBON Lines
### Chart
| Category | begin | end | test |
|---|---|---|---|
| MB051B | 0.187700869565217 | 0.0390589130434782 | 0.302259130434783 |
| MB077B | 0.208258636363636 | 0.0162457575757576 | 0.326233484848485 |
| MB018B | 0.176621916666667 | 0.0349535833333333 | 0.183975333333333 |
| MB011B | 0.577483333333333 | 0.181808260869565 | 0.44725 |
| MB210B | 0.369339275362319 | 0.256052753623188 | 0.44593652173913 |
| MB002B | 0.13717115942029 | 0.0416276086956522 | 0.298708188405797 |
| MB083C | 0.16011 | 0.00201277777777778 | 0.29928 |
| MB310C | 0.124352333333333 | 0.0654234444444444 | 0.258924555555556 |
| MB057B | 0.303439393939394 | 0.0374144696969697 | 0.406843863636364 |
| MB549C | 0.130395666666667 | 0.0268851111111111 | 0.500981333333333 |
| MB543B | 0.199817575757576 | 0.0689602272727273 | 0.287899318181818 |
| MB027B | 0.293455833333333 | 0.0105181060606061 | 0.38529946969697 |
| MB242A | 0.345036527777778 | 0.100640486111111 | 0.310819930555556 |
| MB093C | 0.255667407407407 | 0.106976111111111 | 0.280465555555555 |
| MB082C | 0.190398947368421 | 0.088908947368421 | 0.199428157894737 |
| MB074C | 0.362025 | 0.135920196078431 | 0.303579901960784 |
| MB399B | 0.338061590909091 | 0.0879075757575758 | 0.278351893939394 |
| MB080C | 0.0819718333333333 | 0.005831 | 0.3548675 |
| MB052B | 0.295553083333333 | 0.0808985833333333 | 0.0658650833333333 |
| MB050B | 0.239371794871795 | 0.155035 | 0.166906025641026 |
| MB542B | 0.17619196969697 | 0.100693484848485 | 0.205720606060606 |
| MB112C | 0.25084375 | 0.0957038541666666 | 0.238079791666667 |
| MB298B | 0.307296862745098 | 0.186113333333333 | 0.227181176470588 |
| MB434B | 0.14833847826087 | 0.130816304347826 | 0.525043623188406 |
| MB433B | 0.316002063492063 | 0.168182063492063 | 0.229450873015873 |****
CIbegin
**
**
CIend
*
**
*
**
CItest
**
*
**
****
****
***
**
**
*
*
**
****
***
****
**
*
***
***
**
**
*
*
**
***
**
***
**
**
B
MBON Secondary Lines
### Chart
| Category | | | |
|---|---|---|---|
| MB051C | 0.101067037037037 | 0.00820481481481481 | 0.179459814814815 |
| MB077C | 0.05674 | 0.0 | 0.086703125 |
| MB018B | 0.135579804 | 0.006886667 | 0.383210686 |
| | None | None | None |
| MB011B | 0.471523923858966 | 0.229143333333333 | 0.542297040882544 |
| MB210B | 0.327895169082126 | 0.232557439613527 | 0.534297577189666 |
| | None | None | None |
| MB083C | 0.075944375 | 0.0 | 0.0177063541666667 |
| MB110C | 0.0992203125 | 0.000139166666666667 | 0.126566979166667 |
| | None | None | None |
| MB310C | 0.0325141666666667 | 0.00461291666666667 | 0.471926388888889 |
| MB323B | 0.193837803030303 | 0.0380864393939394 | 0.475082348484848 |CIbegin
*
**
*
**
CIend
***
****
CItest
*
*
****
*
****
*
**
****
****
**
***
*
