# Supplementary material for: Memory Elicited by Courtship Conditioning Requires Mushroom Body Neuronal Subsets Similar to Those Utilized in Appetitive Memory
Source: PLoS One. 2016 Oct 20;11(10):e0164516. doi: 10.1371/journal.pone.0164516 (PMC5072562; doi:10.1371/journal.pone.0164516)
Supplement: S6 Fig — A. Learning index (LI) and memory index (MI) for PAM DAN lines. Expression patterns are directly below the LI and MI for each line. Shading indicates relative levels of expression in each neuron type as reported in (35). Significance is determined using one-sided Wilcoxon signed rank tests with Benjamini-Hochberg post-hoc corrections. *, p < .05; **, p < .01; ***, p < .001; ****, p < .0001. Error bars are SEM, n = 15–24. B. Learning and memory in PPL1 DAN lines. The line identified as a courtship memory hit is boxed in red. Expression patterns are directly below the LI and MI for each line. Shading indicates relative levels of expression in each neuron type as reported in (35). Significance is determined using one-sided Wilcoxon signed rank tests with Benjamini-Hochberg post-hoc corrections. *, p < .05; **, p < .01; ***, p < .001; ****, p < .0001. Error bars are SEM, n = 17–24. (PPTX) [file pone.0164516.s006.pptx]

## Slide 1
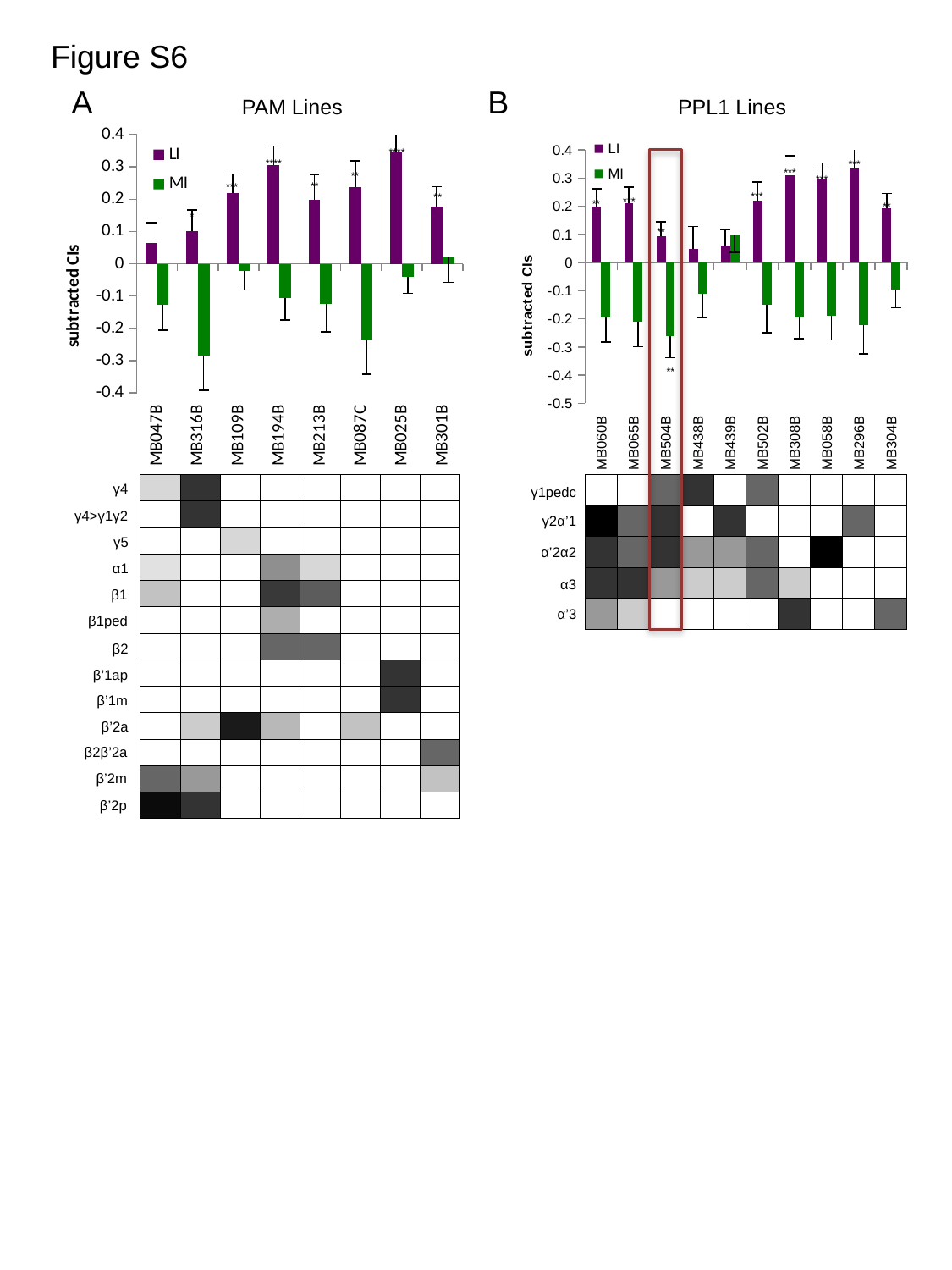

Figure S6
A
B
PAM Lines
PPL1 Lines
### Chart
| Category | | |
|---|---|---|
| MB047B | 0.065386231884058 | -0.127468188405797 |
| MB316B | 0.101137101449275 | -0.283873768115942 |
| MB109B | 0.219717361111111 | -0.0216914583333333 |
| MB194B | 0.306186060606061 | -0.105551212121212 |
| MB213B | 0.198494393939394 | -0.124252196969697 |
| MB087C | 0.236409111111111 | -0.234056888888889 |
| MB025B | 0.344270138888889 | -0.0394618055555556 |
| MB301B | 0.178031875 | 0.0191211805555555 |
### Chart
| Category | | |
|---|---|---|
| MB060B | 0.19740862745098 | -0.193963039215686 |
| MB065B | 0.210003083333333 | -0.209998 |
| MB504B | 0.0926662121212121 | -0.259932121212121 |
| MB438B | 0.0480218421052632 | -0.110648771929825 |
| MB439B | 0.0618110317460317 | 0.0985834126984127 |
| MB502B | 0.220458333333333 | -0.149090486111111 |
| MB308B | 0.310478260869565 | -0.19659847826087 |
| MB058B | 0.294432391304348 | -0.189520579710145 |
| MB296B | 0.333262619047619 | -0.223180396825397 |
| MB304B | 0.193018636363636 | -0.0951790151515152 |****
****
***
***
**
***
**
***
***
**
***
**
**
*
**
**
γ4
| | | | | | | | |
| --- | --- | --- | --- | --- | --- | --- | --- |
| | | | | | | | |
| | | | | | | | |
| | | | | | | | |
| | | | | | | | |
| | | | | | | | |
| | | | | | | | |
| | | | | | | | |
| | | | | | | | |
| | | | | | | | |
| | | | | | | | |
| | | | | | | | |
| | | | | | | | |
| | | | | | | | | | |
| --- | --- | --- | --- | --- | --- | --- | --- | --- | --- |
| | | | | | | | | | |
| | | | | | | | | | |
| | | | | | | | | | |
| | | | | | | | | | |
γ1pedc
γ4>γ1γ2
γ2α’1
γ5
α’2α2
α1
α3
β1
α’3
β1ped
β2
β’1ap
β’1m
β’2a
β2β’2a
β’2m
β’2p
