# Supplementary material for: Memory Elicited by Courtship Conditioning Requires Mushroom Body Neuronal Subsets Similar to Those Utilized in Appetitive Memory
Source: PLoS One. 2016 Oct 20;11(10):e0164516. doi: 10.1371/journal.pone.0164516 (PMC5072562; doi:10.1371/journal.pone.0164516)
Supplement: S7 Fig — A. Courtship indices for the three periods observed, CIbegin, CIend, and CItest, for PAM lines tested in primary screening. Significance is determined using one-sided Wilcoxon signed rank tests with Benjamini-Hochberg post-hoc corrections. *, p < .05; **, p < .01; ***, p < .001; ****, p < .0001. Error bars are SEM, n = 15–24. B. Courtship indices for the three periods observed, CIbegin, CIend, and CItest, for PAM lines tested in secondary screening. Significance is determined using one-sided Wilcoxon signed rank tests. *, p < .05; **, p < .01; ***, p < .001; ****, p < .0001. Error bars are SEM, n = 17–27. C. Courtship indices for the three periods observed, CIbegin, CIend, and CItest, for PPL1 lines tested in primary screening. Significance is determined using one-sided Wilcoxon signed rank tests with Benjamini-Hochberg post-hoc corrections. *, p < .05; **, p < .01; ***, p < .001; ****, p < .0001. Error bars are SEM, n = 17–24. (PPTX) [file pone.0164516.s007.pptx]

## Slide 1
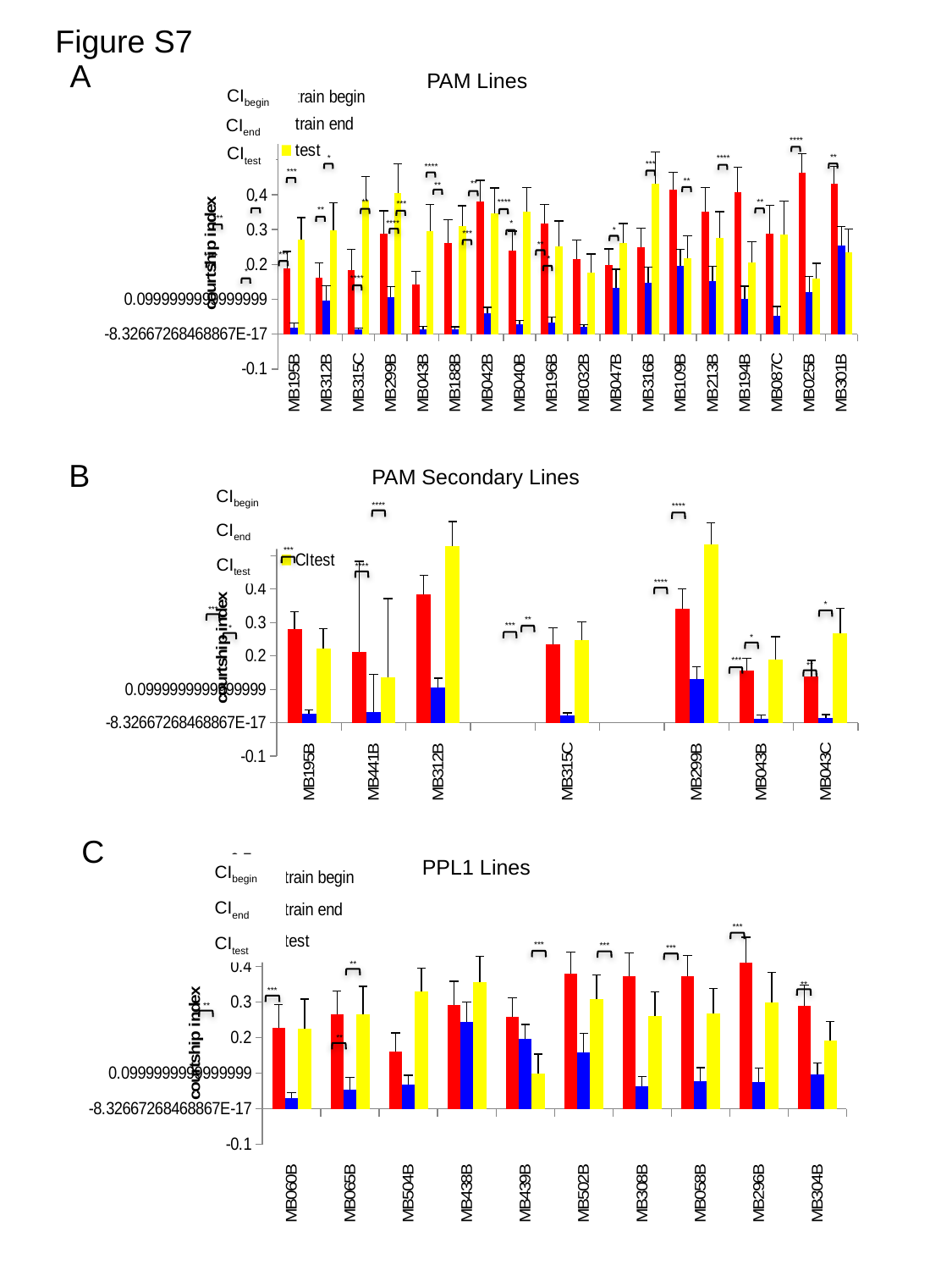

Figure S7
A
PAM Lines
### Chart
| Category | train begin | train end | test |
|---|---|---|---|
| MB195B | 0.188404924242424 | 0.0194399242424242 | 0.270495075757576 |
| MB312B | 0.162604696969697 | 0.0974043181818182 | 0.298619393939394 |
| MB315C | 0.182913157894737 | 0.0124540350877193 | 0.38359850877193 |
| MB299B | 0.28924962962963 | 0.106520277777778 | 0.403469351851852 |
| MB043B | 0.142324848484848 | 0.0142105303030303 | 0.295787121212121 |
| MB188B | 0.262106041666667 | 0.014694375 | 0.309922361111111 |
| MB042B | 0.380740579710145 | 0.0595821739130435 | 0.345789637681159 |
| MB040B | 0.240453484848485 | 0.0271441666666667 | 0.351331212121212 |
| MB196B | 0.318366458333333 | 0.0338386111111111 | 0.252465625 |
| MB032B | 0.214195789473684 | 0.0200991228070175 | 0.176231929824561 |
| MB047B | 0.198603115942029 | 0.133216884057971 | 0.260685072463768 |
| MB316B | 0.249319130434783 | 0.148182028985507 | 0.432055797101449 |
| MB109B | 0.414885208333333 | 0.195167847222222 | 0.216859305555556 |
| MB213B | 0.351205 | 0.152710606060606 | 0.276962803030303 |
| MB194B | 0.407289242424242 | 0.101103181818182 | 0.206654393939394 |
| MB087C | 0.288519333333333 | 0.0521102222222222 | 0.286167111111111 |
| MB025B | 0.463625833333333 | 0.119355694444444 | 0.1588175 |
| MB301B | 0.431045277777778 | 0.253013402777778 | 0.233892222222222 |CIbegin
CIend
****
CItest
**
****
*
***
****
***
**
**
**
*
****
**
**
***
**
***
*
****
*
***
**
***
****
*
*
****
B
PAM Secondary Lines
### Chart
| Category | | | |
|---|---|---|---|
| MB195B | 0.280596228070175 | 0.0275227192982456 | 0.221038859649123 |
| MB441B | 0.212025075757576 | 0.0311253787878788 | 0.137057651515152 |
| MB312B | 0.384429861111111 | 0.105900729166667 | 0.5298925 |
| | None | None | None |
| MB315C | 0.235482283950617 | 0.0217346296296296 | 0.246747716049383 |
| | None | None | None |
| MB299B | 0.342294236 | 0.13007375 | 0.534275833 |
| MB043B | 0.156910175438596 | 0.0121133333333333 | 0.189195175438596 |
| MB043C | 0.137796078431373 | 0.0151566666666667 | 0.267786176470588 |CIbegin
****
****
CIend
***
CItest
****
****
*
***
**
***
*
*
***
**
C
### Chart
| Category | | | |
|---|---|---|---|
| MB060B | 0.227819705882353 | 0.0304110784313725 | 0.224374117647059 |
| MB065B | 0.26489125 | 0.0548881666666667 | 0.264886166666667 |
| MB504B | 0.161653484848485 | 0.0689872727272727 | 0.328919393939394 |
| MB438B | 0.292998684210526 | 0.244976842105263 | 0.355625614035088 |
| MB439B | 0.25963373015873 | 0.197822698412698 | 0.0992392857142857 |
| MB502B | 0.379458194444444 | 0.158999861111111 | 0.308090347222222 |
| MB308B | 0.373737173913044 | 0.0632589130434782 | 0.259857391304348 |
| MB058B | 0.373192898550725 | 0.0787605072463768 | 0.268281086956522 |
| MB296B | 0.409700714285714 | 0.0764380952380952 | 0.299618492063492 |
| MB304B | 0.289352424242424 | 0.0963337878787878 | 0.191512803030303 |***
***
***
***
**
**
***
**
**
PPL1 Lines
CIbegin
CIend
CItest
