# Supplementary material for: Memory Elicited by Courtship Conditioning Requires Mushroom Body Neuronal Subsets Similar to Those Utilized in Appetitive Memory
Source: PLoS One. 2016 Oct 20;11(10):e0164516. doi: 10.1371/journal.pone.0164516 (PMC5072562; doi:10.1371/journal.pone.0164516)
Supplement: S8 Fig — A. Learning index (LI) and memory index (MI) for octopaminergic neuron lines and other extrinsic MB lines. Lines identified as courtship memory hits are boxed in red. Expression patterns are directly below the LI and MI for each line. Shading indicates relative levels of expression as reported in (35). Significance is determined using one-sided Wilcoxon signed rank tests with Benjamini-Hochberg post-hoc corrections. *, p < .05; **, p < .01; ***, p < .001; ****, p < .0001. Error bars are SEM, n = 11–22. B. Courtship indices for the three periods observed, CIbegin, CIend, and CItest, for octopaminergic lines and other MB extrinsic neuron lines tested in secondary screening. Significance is determined using one-sided Wilcoxon signed rank tests with Benjamini-Hochberg post-hoc corrections. *, p < .05; **, p < .01; ***, p < .001; ****, p < .0001. Error bars are SEM, n = 11–22. (PPTX) [file pone.0164516.s008.pptx]

## Slide 1
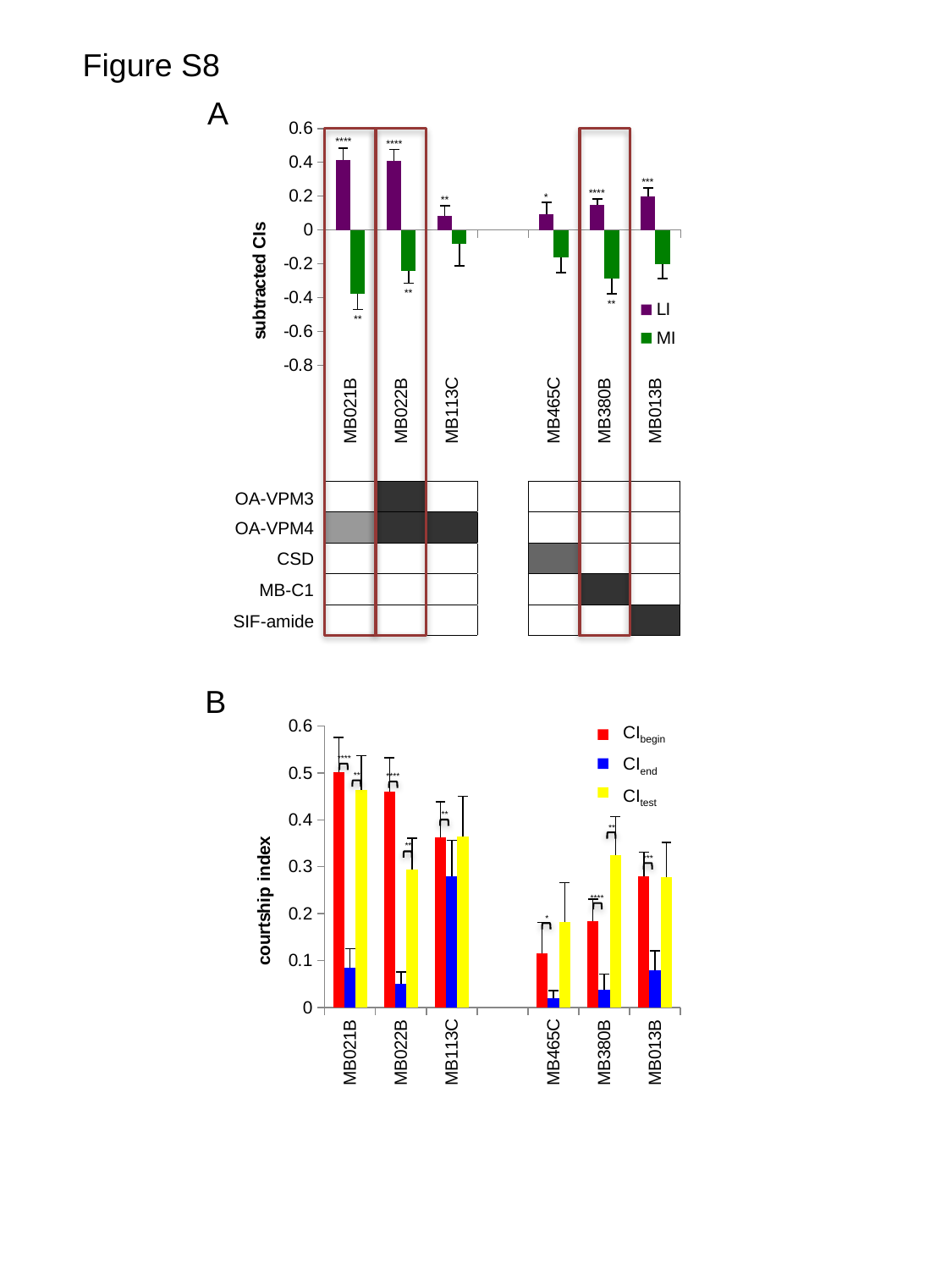

Figure S8
A
### Chart
| Category | | |
|---|---|---|
| MB021B | 0.416235555555556 | -0.379232222222222 |
| MB022B | 0.409088333333333 | -0.243842583333333 |
| MB113C | 0.0832422727272727 | -0.0840168181818182 |
| | None | None |
| MB465C | 0.0945210606060606 | -0.161766818181818 |
| MB380B | 0.14621850877193 | -0.286564912280702 |
| MB013B | 0.200244393939394 | -0.199807954545455 |
****
****
***
****
*
**
**
**
**
OA-VPM3
| | | | | | | |
| --- | --- | --- | --- | --- | --- | --- |
| | | | | | | |
| | | | | | | |
| | | | | | | |
| | | | | | | |
OA-VPM4
CSD
MB-C1
SIF-amide
B
### Chart
| Category | | | |
|---|---|---|---|
| MB021B | 0.500625 | 0.0843894444444444 | 0.463621666666667 |
| MB022B | 0.45958625 | 0.0504979166666667 | 0.2943405 |
| MB113C | 0.363144696969697 | 0.279902424242424 | 0.363919242424242 |
| | None | None | None |
| MB465C | 0.114485151515151 | 0.0199640909090909 | 0.181730909090909 |
| MB380B | 0.18417298245614 | 0.0379544736842105 | 0.324519385964912 |
| MB013B | 0.279038257575758 | 0.0787938636363636 | 0.278601818181818 |CIbegin
****
CIend
**
****
CItest
**
**
**
***
****
*
